# Recent Achievements for Flexible Encapsulation Films Based on Atomic/Molecular Layer Deposition

**DOI:** 10.3390/mi15040478

**Published:** 2024-03-30

**Authors:** Buyue Zhang, Zhenyu Wang, Jintao Wang, Xinyu Chen

**Affiliations:** 1 School of Physics, Changchun University of Science and Technology, Changchun 130012, China; 2 State Key Laboratory on Integrated Optoelectronics, College of Electronic Science & Engineering, Jilin University, Changchun 130012, China; zyw20@mails.jlu.edu.cn; 3 School of Information Engineering, Yantai Institute of Technology, Yantai 264005, China

**Keywords:** atomic layer deposition, functional integration, steric hindrance, thin-film encapsulation, flexible organic light-emitting diodes

## Abstract

The purpose of this paper is to review the research progress in the realization of the organic–inorganic hybrid thin-film packaging of flexible organic electroluminescent devices using the PEALD (plasma-enhanced atomic layer deposition) and MLD (molecular layer deposition) techniques. Firstly, the importance and application prospect of organic electroluminescent devices in the field of flexible electronics are introduced. Subsequently, the principles, characteristics and applications of PEALD and MLD technologies in device packaging are described in detail. Then, the methods and process optimization strategies for the preparation of organic–inorganic hybrid thin-film encapsulation layers using PEALD and MLD technologies are reviewed. Further, the research results on the encapsulation effect, stability and reliability of organic–inorganic hybrid thin-film encapsulation layers in flexible organic electroluminescent devices are discussed. Finally, the current research progress is summarized, and the future research directions and development trends are prospected.

## 1. Background of the Study

### 1.1. Introduction to Flexible Organic Electroluminescent Devices and Their Stability Issues

Organic light-emitting diodes (OLEDs) have now become mainstream products in the display and lighting fields, and advanced electronics industry companies, including Huawei, Samsung, LG, and Sony, have focused on OLED technology in recent years in the product research and development of TVs, tablets, smartphones, and lighting panels [1].

**Figure 1:**
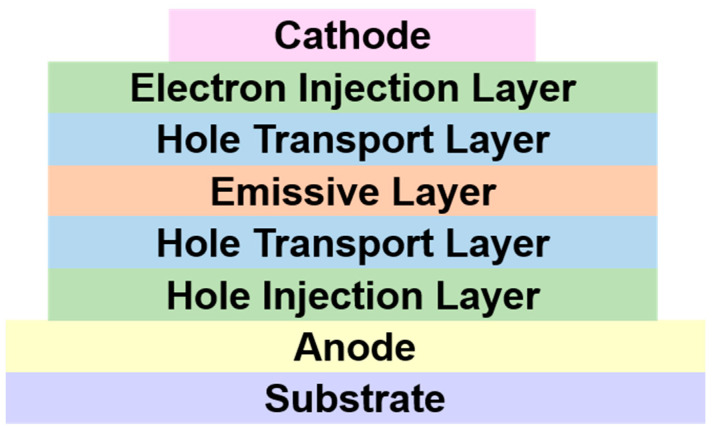
Basic structure of OLEDs.

**Figure 2:**
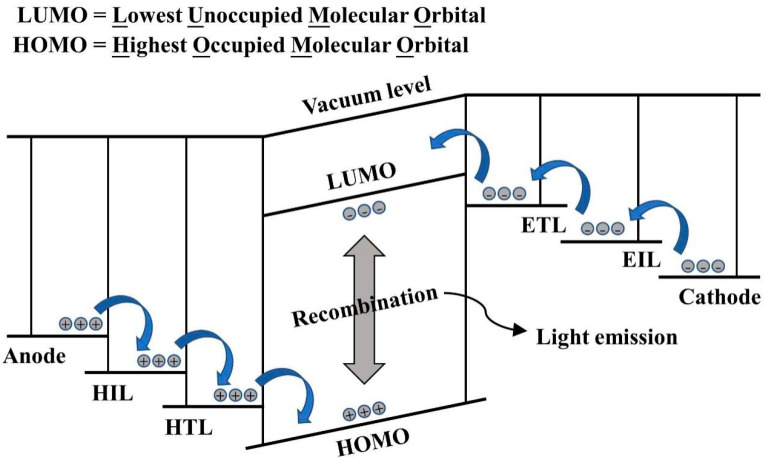
Schematic diagram of energy levels of OLEDs with carrier transport process.

As shown in Figure 1, typical OLEDs often consist of a substrate/anode/hole injection layer/hole transport layer/light emitting layer/electron transport layer/electron injection layer/cathode stacked structure [2]. Electroluminescence is the basic principle of the operation of OLEDs, and Figure 2 depicts the energy band structure of the different functional layers in OLEDs, as well as the carrier transport behavior during operation. Electrons and holes are injected into the organic electron transport layer and the organic hole transport layer through the cathode and anode of the device, respectively, and they move toward each other in their respective transport layers under the action of an applied electric field. They compound to form an exciton in the organic light-emitting layer, and the exciton radiatively jumps back to the ground state and generates light emission, which is emitted from the transparent side of the device [3,4,5].

Compared with liquid crystal display (LCD), OLEDs reduce the backlight module and simplify the design structure [6,7], eliminate part of the light leakage caused by the backlight emission, and realize true all-black displays [8,9]. They also show a thinner and lighter product form [10,11]. In addition, the self-luminous nature of OLEDs results in lower power consumption and excellent stability over a wider temperature range, highlighting the potential for applications in harsh environments [12,13]. Wider color coverage [14] and wider viewing angles shape the product value of OLEDs [15].

In addition to applications in the display industry, OLEDs are also capable of being used in the production of efficient solid-state lighting devices. Compared to traditional inorganic light sources, such as incandescent or fluorescent lamps, OLEDs have higher energy efficiency and light quality while causing zero pollution to the environment [16]. The soft scattered light emitted by OLEDs possesses the characteristics closest to natural light; therefore, OLED light sources are considered to be the ideal light source that is the healthiest and safest [17]. In addition, the large-area feasibility of OLEDs brings great commercial value to them [18].

In recent years, flexible display and lighting technologies have gradually become available, and the wearable and foldable technologies of optoelectronic devices have become the current mainstream research hotspots [19]. In the preparation process, OLEDs can be effectively prepared on substrate materials other than glass substrates, including flexible substrate materials PET, PEN or PI, combined with the excellent mechanical properties of the organic materials themselves, which enables the flexible form of OLEDs to be realized. The technological advantages of OLEDs in the field of display and lighting and the flexibility of the products have made flexible OLEDs become the final form of future display and lighting devices [20,21].

**Figure 3:**
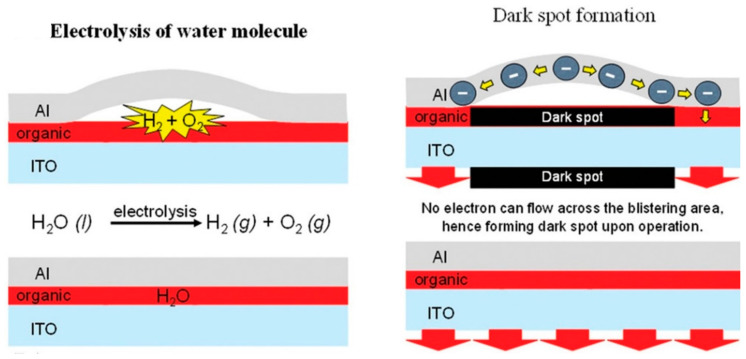
Electrolysis of water molecules and formation of black dots in OLEDs (reproduced from [24] with permission form John Wiley and Sons, 2020).

However, even though OLEDs have many advantages in the industry due to their technological features, their poor environmental stability has been troubling every research team [22,23]. Organic materials and metal electrodes are highly susceptible to degradation and failure upon contact with ambient water vapor. In addition, the ionization reaction between the electrodes and water vapor during operation will cause rapid degradation of the devices. All of these phenomena can lead to the degradation of the performance of OLEDs, or even failure. Figure 3 demonstrates the generation mechanism of black dots in OLEDs, where ambient water vapor fails the organic material while H_2_ and O_2_ generated by the ionization reaction causes a break between the metal electrodes and the organic layer, and carriers are unable to be transported and in the failed portion [24]. In 2018, the research team of H. Fukagawa at the Science and Technology Research Laboratory (STRL) of NHK, Japan, reported a stability study and molecular design scheme for red light OLEDs, and they monitored the change in their luminescence state with time. The device showed a circular failure region under the effect of ambient water vapor, and the area of the region gradually became larger with the increase in the time [25].

**Figure 4:**
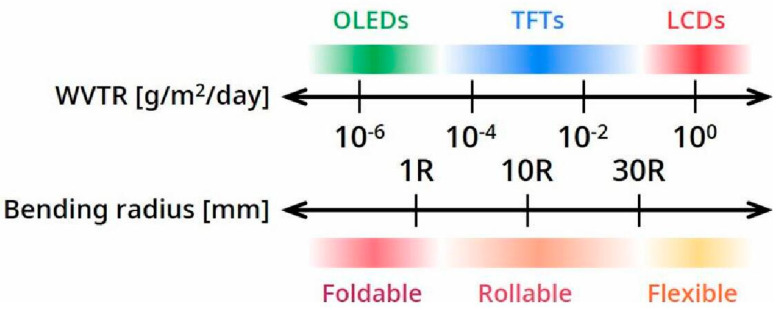
Water vapor barrier performance and mechanical property requirements of optoelectronic devices for different application scenarios (reproduced from [28] with permission from Taylor and Francis).

Therefore, in order to enhance the operational stability of OLEDs, effective protection means are often used to isolate the device from environmental water vapor, avoid direct contact, and minimize device damage [24]. Among them, encapsulation technology is currently one of the most effective means of protection for organic optoelectronic devices, with a high-performance barrier layer covering the outer surface of the device, thus forming a permeable barrier to environmental water vapor [26,27]. The current academic and industrial consensus is that the water vapor transmittance rate (WVTR) of the barrier layer reaches 10^−6^ g/m^2^/day, which is the basic condition to ensure the 10-year service life of organic optoelectronic devices. The standard applies to both rigid and flexible devices, and the water vapor barrier performance of different optoelectronic devices and the mechanical properties are shown in Figure 4 [28].

### 1.2. Thin-Film Packaging Technology and Its Flexible Applications

**Figure 5:**
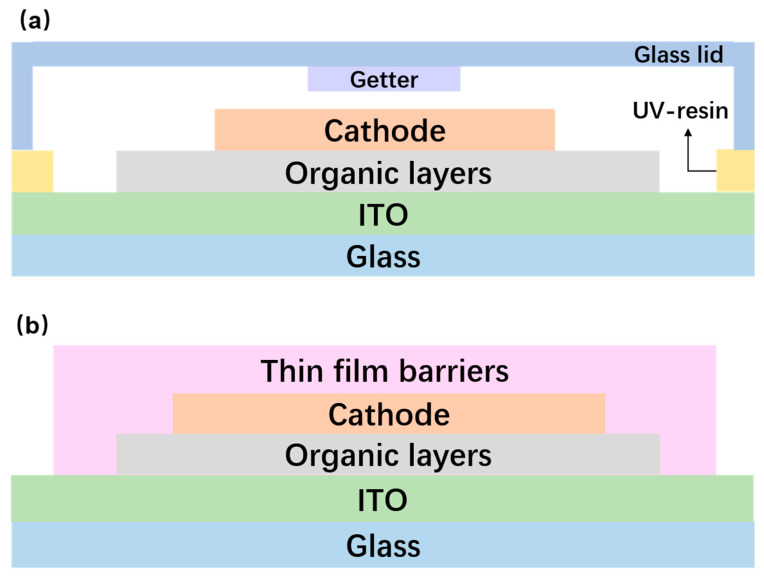
(**a**) Schematic diagram of glass cover package. (**b**) Schematic diagram of thin-film package.

Encapsulation technology is an effective means to avoid OLEDs being damaged by environmental water vapor [29,30,31]. As shown in Figure 5a, the traditional OLED encapsulation process is realized by the cavity structure cover plate encapsulation by the glass cover plate. Between the substrate and the glass cover plate, the epoxy resin was used to seal the surrounding area. The entire encapsulation process was completed in the inert gas (such as argon, nitrogen, etc.) within the atmosphere, and the desiccant (such as calcium oxide, etc.) was placed in the cavity to minimize the water vapor [32,33,34]. The glass cover shows excellent protection performance in the early stage for organic optoelectronic devices and is widely used in various optoelectronic devices. However, the introduction of glass covers has significantly increased the overall weight of the devices, hindering the development of lightweight optoelectronic devices [35,36,37]. In addition, the physical properties of the glass cover make it difficult to realize the flexible form of OLEDs, and the three-dimensional mesh structure formed after the curing of the epoxy resin, which is prone to generating large internal stresses. Thus, the brittleness of the cover increases, and it can even crack or other damages can form in a short period of time. The cracks would provide a penetration path for the ambient moisture, resulting in the decline of the sealing performance. The desiccant in the sealing structure will swell after absorbing water vapor, affecting the encapsulation performance, and the protection efficiency of the cover encapsulation is reduced [26]. The iteration of display technology, from flat panel display, curved display, and then to the future of the curly, foldable display technology, means that the packaging technology should also be gradually flexible. Therefore, the disadvantages of the cover package technology are obvious, and it is a big obstacle to the process of lightweighting and flexibilization of optoelectronic devices, which makes its replacement by flexible packaging technology an inevitable trend for the future development of optoelectronic devices.

At present, thin-film encapsulation technology, as an advanced encapsulation process, has been gradually applied to various types of optoelectronic devices [38]. Inorganic thin films, such as SiNO, Al_2_O_3_, ZrO_2_, etc., prepared using chemical vapor deposition (CVD) have undergone a series of encapsulation-related studies, and the obtained encapsulation models can meet the current application requirements.

As shown in Figure 5b, in addition to rigid devices, thin-film encapsulation technology can also be used to efficiently encapsulate and protect flexible OLEDs prepared based on flexible substrate materials (e.g., PET, PEN, PES, PI, etc.), which ensures that the devices are bendable and foldable, protecting the devices from damage brought about by the intrusion of ambient water vapor [39]. The encapsulated thin-film material is grown directly on the surface of the device, eliminating the edge penetration effect caused by the sealing process. The development and widespread use of thin-film encapsulation technology is a key step in the lightweighting process of flexible displays. Thin-film encapsulation technology can be divided into inorganic thin-film encapsulation technology, organic thin-film encapsulation technology, organic/inorganic hybrid thin-film encapsulation technology, and so on, according to the type of composition of the encapsulated thin-film material.

**Figure 6:**
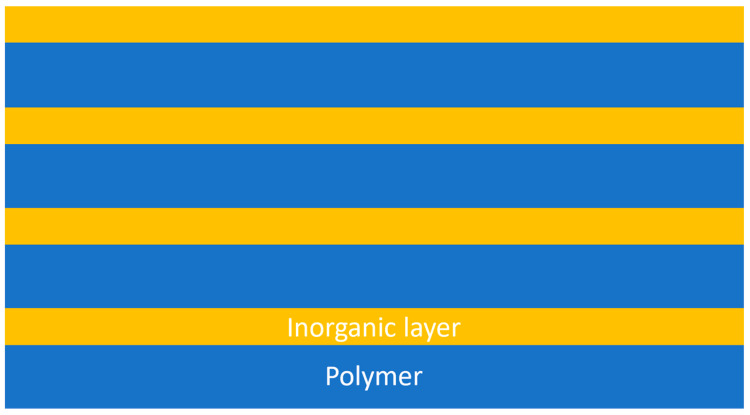
The image of Barix organic–inorganic stacked encapsulated film cross-section.

In order to make thin-film encapsulation technology meet the water vapor barrier needs of organic optoelectronic devices, many advanced optoelectronics industry companies and research institutes have invested a great deal of money in the development of more advanced fabrication processes and encapsulation structures. In 2002, The company “Vitex Systems” developed a new flexible OLED encapsulation technology named “Barix” [40], shown in Figure 6. The technology employs a combination of UV-cured organics and sputtered inorganics to grow organic and inorganic films alternately on the surface of the protected optoelectronic device to form an organic/inorganic stacked-film structure. The water vapor barrier performance of single-layer inorganic films is limited by the water vapor permeation path generated by defects within the film, while in the Barix encapsulation structure, the introduction of the organic film sandwich structure can effectively couple the defects in the adjacent inorganic film to extend the water vapor permeation path, which will have a greater improvement in the water vapor barrier performance of the film. In 2011, South Korea’s Samsung purchased a patent for the technology and collaborated with the Korea Institute of Technology KAIST to create the organic/inorganic stacked-film structure. In 2011, Samsung purchased the patent and cooperated with KAIST to apply this encapsulation system to the protection of OLEDs. By using multilayer thin-film wrapping encapsulation on substrate and the surface of OLED devices, forming alternating dense inorganic water vapor barrier layers with organic polymers in a vacuum environment, with the total thickness of the stacked structure of about 3 μm. The thin-film encapsulation process can directly grow thin films on the surface of OLEDs, avoiding the use of other encapsulation materials or mechanical encapsulation components, reducing the size and weight of the device, and minimizing the damage of the device caused by the environmental water vapor infiltration. Barix encapsulation structure embodies the excellent encapsulation performance, which can be effectively applied to flexible displays. However, the performance of OLEDs will be degraded by high-temperature and ultraviolet radiation [41]; in addition, the Barix encapsulation scheme is cumbersome in the growth process, the process flow is slow and the cost is high, which cannot fully meet the demand of the future device flexibilization process, and it has been gradually abandoned by the industry.

CVD technology is a process of thin-film growth by chemical reaction on the substrate surface in a vacuum environment, and the short process time as well as the high densities of the prepared films have led to the increasing application of CVD technology in the preparation of inorganic barrier layers in thin-film encapsulation processes [42]. However, to obtain a sufficiently dense thin-film material by CVD technology that can be effectively applied in the thin-film encapsulation of organic optoelectronic devices, the required process temperature often exceeds 150 °C [43]. OLEDs are a kind of electronic device that are sensitive to the process temperature, and higher process temperatures can degrade the organic functional materials [44], affecting the performance of the device, thereby hindering the development of CVD technology in the field of thin-film packaging. Plasma-enhanced chemical vapor deposition (PECVD) utilizes plasma to compensate for the problem of low reactivity caused by reactive precursors or process temperature [45,46], which makes the thin-film vapor growth process no longer limited by the process temperature and realizes the high quality of the thin-film growth process under low process temperature. The introduction of plasma enabled PECVD technology to meet the process requirements for the thin-film encapsulation of OLEDs, and its related encapsulation process link is practical to this day. In 2008, Kateeva, founded by C. Madigan et al. of MIT, proposed inkjet printing (IJP) technology [47]. The growth of thin-film materials is achieved by directly spraying an ink containing the corresponding nano-composition on a flexible or rigid substrate [48,49]. Since the ink-spraying path during the IJP process can be set by a software program, it is possible to prepare patterned thin-film materials without the help of a mask plate, which is a promising thin-film printing technology [48]. The IJP process has perfected the encapsulation structure of inorganic thin films, which makes the thin-film encapsulation technology truly applicable to the protection of optoelectronic devices, and the organic–inorganic stacking of PECVD/IJP/PECVD has been used for the protection of optoelectronics. PECVD organic–inorganic stacked encapsulation structure is also one of the most effective means of encapsulation for current devices [49], but the method requires a thicker organic layer coupled with the PECVD film, so that the position of the outer inorganic film is far from the neutral plane and generates a large surface deformation during the bending process, and the encapsulated film surface stresses can easily reach the modulus of rupture of its counterpart, at the expense of the mechanical properties of encapsulated films.

In summary, looking through the development path of the thin-film encapsulation process, with the iteration of display technology, the corresponding thin-film encapsulation process is also gradually moving towards high efficiency and advanced. Facing the future development plan of foldable and rollable display technology, the current thin-film encapsulation technology will not be able to continue to meet the future demand for the corresponding flexible applications. PECVD/IJP/PECVD encapsulation structure needs to prepare thicker film material to couple the internal defects of the film in order to achieve excellent encapsulation performance, at the expense of the mechanical properties of the encapsulated film. At present, many advanced optoelectronics companies combined with scientific research units have begun to look for new thin-film encapsulation processes, which will determine the arrival of the next generation of optoelectronic devices.

### 1.3. Introduction to Atomic Layer Deposition

Similar to CVD, atomic layer deposition (ALD) is a thin-film preparation technique based on chemical reactions on the substrate surface, and, in addition to similar film growth conditions, some precursor materials are common between the two processes [50]. The difference is that the CVD technique maintains the coexistence of the two precursor materials in a vacuum reaction chamber, which chemisorb on the substrate surface to form thin films [51]. In contrast, the surface chemical reaction established by ALD technology occurs independently and alternately for each precursor material, and each precursor material has a self-limiting reaction property, and the corresponding self-limiting surface half-reaction grows the material layer by layer on the substrate surface in the form of a single atomic layer [52], and the successive self-limiting surface reactions satisfy the need for single-atomic-layer control and co-conformal deposition in the process of thin-film growth.

**Figure 7:**
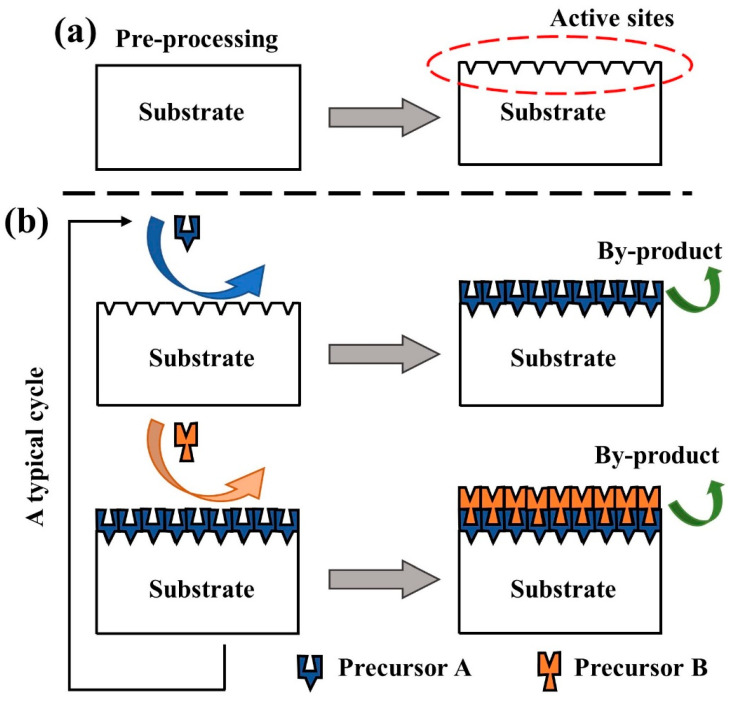
(**a**) Substrate surface pretreatment process. (**b**) Schematic of the ALD binary reaction sequence realized using precursors A and B for self-limiting surface reaction.

The surface reaction process of ALD technology is characterized by continuous, self-limiting properties [53]. As shown in Figure 7, a typical ALD process tends to employ a binary reaction sequence for film growth, where two precursors complete their respective corresponding half-reactions in sequence on the substrate surface to achieve a monolayer deposition process of a binary compound film. The active sites on the substrate surface are the basis for ALD film growth [54]; therefore, before the film growth process starts, the substrate is often introduced with active sites, or it increases the active site density by means of some surface pretreatment [55]. For example, the number of hydroxyl groups (-OH) on the substrate surface can be greatly enhanced by oxygen plasma (O_2_ plasma) or UV radiation, as shown in Figure 7a. The binary reaction sequence involved in the ALD process is divided into four steps, as shown in Figure 7b. First, precursor A is passed into the reaction chamber to undergo a self-limiting surface reaction with the active sites on the substrate surface, adsorbing a monoatomic layer and generating the corresponding by-products, followed by purging the entire chamber and piping using inert gas Ar to evacuate the residual precursor A and reaction by-products. Next, precursor B is passed into the reaction chamber and undergoes a self-limiting surface reaction with the active sites provided by precursor A, adsorbing another monatomic layer, and is accompanied by the production of by-products. Finally, Ar is again used as a purge gas to evacuate the residual precursor B with the corresponding by-products, and the re-exposed active sites are able to react with precursor A again. At this point, a cycle ends and the growth of a layer of product is completed. Repeat the above cycle N times and customize the ALD process parameters according to the usage requirements.

Since the number of active sites on the substrate surface is finite, the amount of surface material is deposited via the half-reactions, corresponding to each surface half-reaction having its own saturation state [56]. If both of the independent surface half-reactions are self-limiting, the two reactions can be carried out in a sequential, alternating manner to obtain a layer-by-layer deposition process of thin films and to satisfy atomic-level controllability [57]. The ALD process is governed by a surface chemistry reaction, and since its surface reactions are carried out in a sequential, alternating manner, the two precursors do not come into contact in the gas phase, and the separation would suppress the possible occurring CVD-like gas-phase reactions [58], avoiding the appearance of particulate products on the film surface.

Although precursor materials have self-limiting reaction properties, the reactions at the surface active sites are sequential due to different precursor gas fluxes. Precursors may physically adsorb in the form of van der Waals forces to the surface where the reaction has been completed and subsequently desorb from that region to continue to react with other unreacted surface regions and produce conformal deposition [59]. Because the ALD technique avoids the stochastic nature of the precursor flux, the self-limiting nature of the surface reactions also produces non-statistical deposition, which results in each surface half-reaction being driven to occur and reach near saturation. Therefore, ALD-grown films are very smooth and conformal to the original substrate [60]. The films tend to be continuous and pinhole-free because there are almost no surface active sites remaining during film growth [61]. This property is important for the preparation of excellent dielectric and water vapor barrier films.

Currently, ALD technology has great application prospects in the preparation of ultrathin and ultrafine films. Typical thin-film materials, such as Al_2_O_3_, SiO_2_ and ZnO, have been used in various electronics industries [62,63,64,65]. In recent years, thin-film deposition and component modulation have been widely used in micro/nanofabrication technologies, such as mechanical structures, electrical isolation and connectivity [66,67,68,69]. The International Technology Roadmap for Semiconductors (ITRS) applies ALD technology to the preparation of high dielectric constant gate oxides in MOSFET structures and copper diffusion barrier layers in back-end interconnects [70]. The miniaturized layout of semiconductor processes and the resulting high depth-to-width ratio structure of the products make the precise control and conformal coating of thin-film deposition techniques a key technological need, and the ALD process provides an effective solution to this need.

In addition, due to the excellent densification of the film grown using ALD technology, it can form a good gas molecule barrier within a hundred nanometer thickness [71], and the ultrathin film morphology provides an important technical support for the application of flexible products. Therefore, the current ALD technology is widely recognized as one of the effective means of protecting optoelectronic devices in the future, and ALD-based thin-film encapsulation technology demonstrates a thinner and lighter encapsulation weight and superior flexibility compared to existing encapsulation means [72]. Prof. S. F. Bent of Stanford University believes that ALD will become an effective solution to the problem of thin-film packaging due to its precise and controllable atomic scale film growth [73]. At present, inorganic materials such as Al_2_O_3_, ZrO_2_, SiO_2_, HfO_2_, etc., prepared using ALD technology have been subjected to a lot of research and achieved excellent encapsulation results [74,75,76,77,78,79,80]. However, thin-film encapsulation materials based on ALD technology are usually dominated by oxides, with stable binary bonds between metal and oxygen atoms in the molecular structure, leading to high Young’s modulus for oxide films, which tend to be rigid as the density and thickness of the films increase [81]. In addition, plasma-assisted ALD technology (plasma-enhanced atomic layer deposition, PEALD) is often employed to compensate for the lack of low-temperature reactivity in order to meet the demand for low-temperature deposition [82,83,84]; however, the introduction of O_2_ plasma introduces a large amount of residual stresses inside the films [85]. The inherent properties attributed to ALD-grown inorganic materials, such as low ductility, low fracture toughness, and high brittleness, limit the durability and reliability of inorganic encapsulation materials during mechanical motions [86,87,88,89,90,91], and the inorganic films are unable to maintain the encapsulation stability under rigorous mechanical motions, despite the excellent encapsulation properties.

Similar to the ALD technique, the molecular layer deposition (MLD) technique is capable of depositing single molecular layers layer-by-layer onto the substrate surface, and it is often used for the growth of organic or organic–inorganic hybrid materials [92,93]. It is worth noting that MLD techniques often have some organic components introduced, and the organic or organic–inorganic hybrid films prepared using them have excellent mechanical properties [94,95]. However, MLD often uses organic precursors as the surface growth unit of the monomolecular layer, which contains long-chain organic structures. This will lead to a large molecular size of the precursor material, which tends to form spatial site resistance on the substrate surface during the semi-reaction process and obscures some of the active sites, and thus limits the degree of saturation [96,97], and the residual active sites give rise to a higher number of defective states inside the film. The defective sites then have the opportunity to provide penetration paths for ambient water vapor, which greatly affects the water vapor barrier performance of the film [98,99]. In addition to this, during the mechanical movement of the film, additional stresses are concentrated at the film defect locations [100,101], and location-specific film stress release patterns are thus induced to occur. Whether it is inorganic or organic materials, using either one of them alone cannot meet the future packaging needs of flexible optoelectronic devices.

At present, domestic and foreign research teams and advanced optoelectronics enterprises hold the organic/inorganic stacked packaging structure as one of the key research directions, the excellent packaging characteristics of inorganic materials combined with the mechanical properties being brought about by organic materials, so that the organic/inorganic stacked packaging structure has become the current mainstream packaging program, supporting the most advanced thin-film packaging technology. In the future, in the face of foldable, wearable and other ultra-flexible optoelectronics, the innovation of thin-film packaging technology is bound to come soon.

CVD, ALD and MLD are effective film deposition technologies; they all own the advantages of uniform and conformal deposition, which allows for the deposition of thin films with excellent uniformity over complex and irregularly shaped surfaces, making them suitable for applications even on three-dimensional structures. That said, the ALD and MLD are limited by the slow deposition speed, which indicates the limited application scenarios. The CVD has been applicated in commercial encapsulation, while the fabrication may destroy the devices, and the particles would form during the deposition process.

## 2. Research History and Current Status of Development

ALD technology is an emerging film preparation technology. Due to its preparation of dense and conformal film, the lower film thickness can be achieved with the same level of water vapor barrier performance compared with other film preparation methods. Therefore, ALD technology has greater prospects for development in the field of flexible thin-film packaging, and it will be a favorable means of overall device thinning at the packaging level. At present, ALD technology in the field of thin-film encapsulation is mainly used to prepare highly dense inorganic encapsulation films, but the mechanical properties of ALD inorganic films are often poor. In order to realize the flexible encapsulation, it is highly desirable to introduce organic components to enhance the mechanical properties of the encapsulation structure. In this thesis, the research progress and status of the ALD process in the field of thin-film encapsulation will be introduced from the aspects of “inorganic encapsulation materials”, “organic and inorganic composite encapsulation materials”, and “flexible encapsulation structure design”.

### 2.1. Analysis of Inorganic Thin-Film Encapsulation Materials and Their Properties

**Figure 8:**
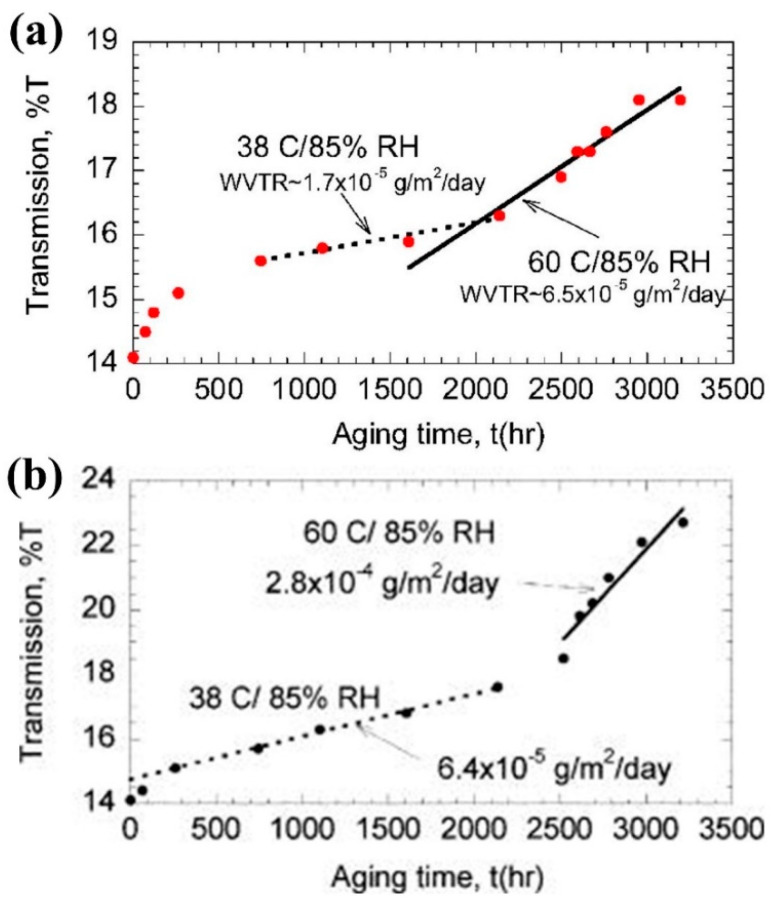
Optical transmittance curves with time for calcium test elements in different test environments with encapsulated films of (**a**) 25 nm Al_2_O_3_ and (**b**) glass cover (reproduced from [102] with permission from AIP Publishing).

The inorganic encapsulation layers involved in ALD technology include Al_2_O_3_, SiO_2_, ZrO_2_, etc. Each of these inorganic materials possesses excellent densification, high conformality, and precise thickness control during the growth process. Among them, Al_2_O_3_, as the most typical and mature ALD inorganic material, is often used in the packaging of optoelectronic devices. In 2006, P.F. Carcia’s research team at DuPont Research Institute’s Development Experiment Station utilized ALD technology to grow a 25 nm layer on a PEN substrate by selecting H_2_O and Trimethylaluminum (TMA) as the oxidizing agent and aluminum source, respectively. A 25 nm thick Al_2_O_3_ film was grown on a PEN substrate, and the barrier properties of the film were quantitatively analyzed by calcium testing. The calcium electrode was isolated by the encapsulation layers. With the penetration of oxygen and moisture, the color of calcium electrode was changed. By measuring the variation of transmission, the WVTR could be estimated. The optical transmittance curves of the calcium-tested components are shown in Figure 8.

It was found that the ambient temperature affects the water vapor barrier performance of encapsulated films. When the ambient humidity was maintained at 85% and the ambient temperature was set at 38 and 60 °C, the WVTR of the PEN/Al_2_O_3_ films exhibited 1.7 × 10^−5^ g∙m^−2^∙day^−1^ and 6.5 × 10^−5^ g∙m^−2^∙day^−1^, respectively. After the test conditions were maintained unchanged, and the ALD-Al_2_O_3_ film was replaced with a glass cover sheet and fixed by epoxy resin, the WVTR was increased. The research team was surprised by this result but did not give an explanation for it. A more reasonable explanation would be that the epoxy resin provides a penetration path for water vapor in this harsh test environment. The glass itself has excellent vapor barrier properties, but the epoxy resin joiner reduces the overall performance. On this basis, the research team estimated the WVTR of 25 nm thick ALD-Al_2_O_3_ film at 23 °C to be up to 6.5 × 10^−5^ g∙m^−2^∙day^−1^ based on the apparent activation energy theory, which is close to the water vapor barrier performance of the glass cover [102].

In 2016, H. Jeon’s research team at Hanyang University grew 50 nm thick Al_2_O_3_ on the surface of PEN substrates using ALD technology, and the film exhibited a WVTR of 3 × 10^−3^ g∙m^−2^∙day^−1^ with suppressed the generation of black dots in flexible OLEDs [103]. In 2019, H. Kim’s research team at Yonsei University in South Korea centered on the PEALD–SiO_2_ film growth behavior on the surface of PEN substrate and found that the pretreatment of PEN substrate using O_2_ plasma can enhance the adhesion between inorganic film and substrate, as well as reduce the difficulty of the film growth process of PEALD technology on the surface of flexible substrate [78].

At present, many research teams have grown inorganic films on the surface of flexible substrates using ALD technology and have made the evaluation and optimization of film quality. At the same time, the flexible devices protected by inorganic films also show improved stability. Although these works do not take into account the mechanical properties of the film, it is still a good practice and validation of the application of ALD technology in the field of flexible packaging.

**Figure 9:**
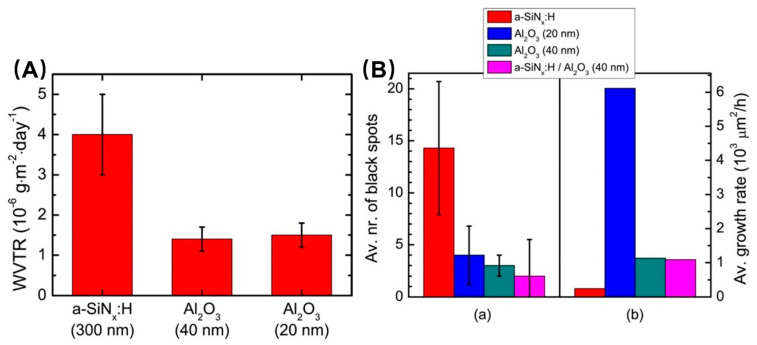
(**A**) Comparison of the water vapor barrier properties of a-SiN_x_:H grown with a thickness of 300 nm by PECVD and Al_2_O_3_ films grown with a thickness of 20 nm versus 40 nm by PEALD in a test environment of 20 °C and 50% relative humidity for 70 days. (**B**) Average of OLEDs encapsulated with different barrier layers (a) and (b) in a 114-day aging experiment number of black dots generated and average black dot growth rate (reproduced from [104] with permission from American Vacuum Society).

CVD technology, as a traditional inorganic film growth process, is undoubtedly the biggest competitor of ALD technology in the field of thin-film encapsulation. In order to explore the differences in encapsulation performance between the two, in 2012, the research team of W.M.M. Kessels, Eindhoven University of Technology, carried out a comparative study on the encapsulation performance of two SiN_x_:H and Al_2_O_3_ encapsulated thin films, which were prepared using PECVD and PEALD, respectively [104]. As shown in Figure 9a, both 20 nm and 40 nm thick PEALD-Al_2_O_3_ films have a WVTR of less than 2 × 10^−6^ g∙m^−2^∙day^−1^ at 20 °C and 50% relative humidity (RH), while the 300 nm thick PECVD-SiN_x_:H only exhibits 4 × 10^−6^ g∙m^−2^∙day^−1^ WVTR in the same environment. Although the thickness of PECVD-SiN_x_:H (300 nm) is about an order of magnitude higher than that of PEALD- Al_2_O_3_ (40 nm or 20 nm), the latter is still far ahead in terms of water vapor barrier performance. The team then encapsulated the OLEDs in four different structures: 300 nm PECVD-SiN_x_:H, 20 nm PEALD-Al_2_O_3_, 40 nm PEALD-Al_2_O_3_, and 300 nm PECVD-SiN_x_:H/40nm PEALD-Al_2_O_3_ stack. The encapsulated devices were placed in an environment of 20 °C and 50% RH for aging tests to observe the evolutionary behavior of the black spots on the organic materials produced by ambient water vapor with aging time. As shown in Figure 9b, after 114 days of aging treatment, the black dot density on the surface of the PECVD-SiN_x_:H encapsulated OLEDs is higher, indicating that the encapsulated film has more internal defects, and multiple water vapor penetration paths are formed. This performance comparison work demonstrates the technical superiority of PEALD technology in the encapsulation field.

Inorganic nanostacked structures exhibit superior water vapor barrier properties compared to single-layer inorganic films. In 2009, T. Riedl’s team at the University of Technology Braunschweig suggested that alternating nanostacked structures can effectively inhibit the formation of microscopic pores and nanocrystals, which leads to a significant reduction in the probability of the occurrence of water vapor infiltration pathways [105]. In 2012, S.M. Cho’s team at Sungkyunkwan University used ALD technology to prepare Al_2_O_3_/ZrO_2_ nanostacked structures with a 350% improvement in water vapor barrier properties compared to the same thickness of inorganic films, and they pointed out that the ZrAl_x_O_y_ structure at the interface of the film was able to block the water vapor infiltration paths and that the water vapor barrier performance of the film improved with the increase in the number of stacked layers [106]. In 2014, the team of Pohang University of Science and Technology’s C. E. park’s team pointed out that the amorphous Al_2_O_3_ film is prone to degradation under the action of water vapor, and its WVTR exhibits 3.75 × 10^−4^ g∙m^−2^∙day^−1^; whereas the TiO_2_ film possesses excellent corrosion-resistant passivation properties, and the Al_2_O_3_/TiO_2_ nano-stacked layer structure effectively inhibits the degradation and reduces the WVTR to 1.81 × 10^−4^ g∙m^−2^∙day^−1^ [107]. And, subsequently, the team grew dense and amorphous Al_2_O_3_/HfO_2_ thin films using ALD technology in 2017 and showed excellent chemical stability, which exhibited a WVTR of 6.75 × 10^−6^ g∙m^−2^∙day^−1^, compared to Al_2_O_3_ monolayers (WVTR = 3.26 × 10^−4^ g∙m^−2^∙day^−1^), the WVTR decreased by two orders of magnitude, again demonstrating the enhanced water vapor barrier performance afforded by nanostacked films [74].

Although the inorganic films prepared using ALD technology can exhibit excellent water vapor barrier properties in monolayer or nanostacked structures, the ALD film growth process is susceptible to the process temperature, and process temperature can affect the film quality and degrade the water vapor barrier properties either too low or too high [108]. Each ALD film growth process has its corresponding temperature window, and when the process temperature is within the temperature window, the film quality is excellent and almost does not change with the process temperature, but once the process temperature jumps out of the temperature window, the film quality is temperature modulated [109,110]. When the process temperature is too high, the saturation chemisorption ratio of precursor molecules on the substrate surface decreases; when the process temperature is too low, the condensation of precursor molecules on the substrate surface increases, triggering surface CVD reactions. As a result, when the process temperature is not within the temperature window, the number of film defects increases, inducing a large number of water vapor penetration paths and a consequent decrease in water vapor barrier performance.

**Figure 10:**
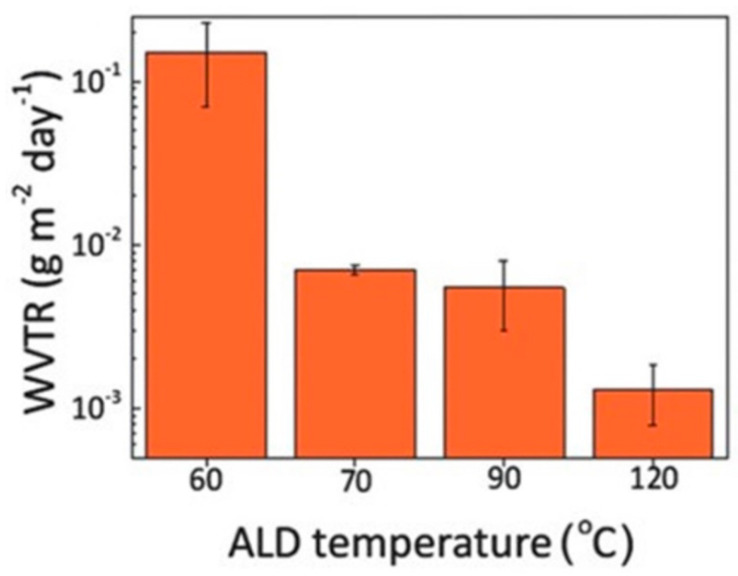
WVTR of ALD-Al_2_O_3_ thin films at different growth temperatures (reproduced from [42] with permission from John Wiley and Sons).

In 2017, S.G. Im’s research team at the Korea Advanced Institute of Science and Technology (KAIST) grew 21.5 nm thick Al_2_O_3_ thin films by using the thermal ALD (Thermal ALD, T-ALD) technique, using H_2_O and TMA as the oxidant and aluminum source, respectively. They investigated the effects of different process temperatures (60 to 120 °C) on the water vapor barrier properties of the films. As shown in Figure 10, the water vapor barrier performance of the films gradually increased with the gradual increase in the process temperature from 60 to 120 °C, corresponding to a gradual decrease in the WVTR from the order of 10^−1^ g∙m^−2^∙day^−1^ to the order of 10^−3^ g∙m^−2^∙day^−1^. The research team concluded that the growth of inorganic thin films is achieved through the chemical reaction of reactive substances adsorbed on the substrate surface, and the decrease in substrate temperature inhibited the chemical combination between surface TMA and H_2_O. The unreacted residue was inside the Al_2_O_3_ films, leading to the increase in the film defect density, and thereby the water vapor penetration path, and decreasing the water vapor barrier performance [42].

**Figure 11:**
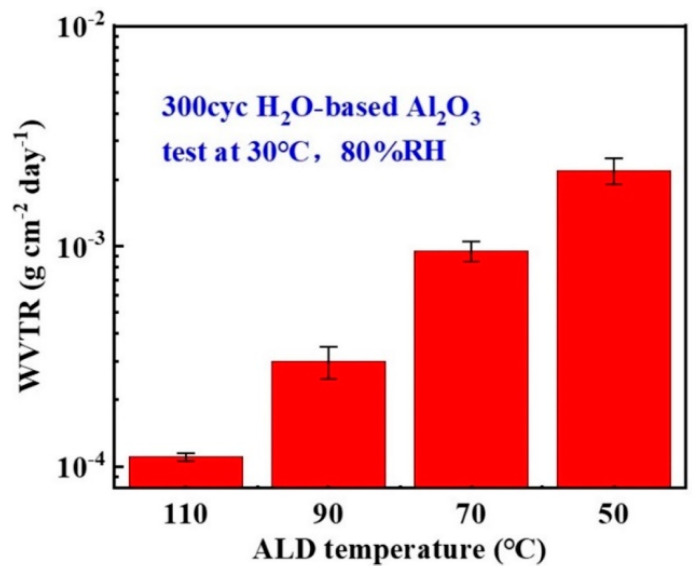
WVTR of Al_2_O_3_ thin films grown using T-ALD technique at different growth temperatures. The ALD temperature represents the reaction temperature (reproduced from [111] with permission from Elsevier).

In addition to this, the authors had carried out similar experiments using process temperature as a research variable in their research in 2020, during which H_2_O and TMA were selected as the oxidant and aluminum source, respectively, and 45 nm thick Al_2_O_3_ thin films were prepared using the T-ALD technique at process temperatures ranging from 50 to 110 °C. Subsequently, the electrical calcium test was utilized to measure the film’s WVTR, and the results of the study are shown in Figure 11. The WVTR of the Al_2_O_3_ films exhibited 2.2 × 10^−3^ g∙m^−2^∙day^−1^ when the process temperature was 50 °C, while the WVTR of the Al_2_O_3_ films exhibited 1.1 × 10^−4^ g-m^−2^∙day^−1^ when the process temperature was 110 °C. The increase in the process temperature from 50 to 110 °C decreased the WVTR of the films by one order of magnitude, and the water vapor barrier performance was significantly improved [111], and this trend is consistent with the results obtained by S. G. Im’s research team.

In addition to the limitation of process temperature on film quality, the poor mechanical properties of inorganic films have been a key issue limiting their application in flexible packaging, such as low ductility, low fracture toughness, and high brittleness of the films, all of which limit the durability and reliability of the films when they are subjected to mechanical stresses or deformation [86,87,88,89,90,91].

The poor mechanical properties of inorganic thin films are attributed to the inherent properties of the material, the growth conditions, and the interaction between the film and the substrate. For example, some inorganic oxide or nitride materials lack the ability to move dislocations due to the strong ionic bonding structure contained within them, resulting in low fracture toughness and ductility. In addition, inorganic thin-film materials are often grown using physical vapor deposition (PVD) or CVD technology; these growth processes will introduce defects and residual stresses inside the film. In the process, the film is subjected to mechanical movement, the defects will lead to the concentration of the film stress, when the load stress and the accumulation of residual stresses in the film accumulate, and the defect location will preferentially produce fracture or delamination to release the stress, resulting in film damage, which leads to further degradation of the mechanical properties of the film. In 2011, S.M. George’s research team analyzed the mechanical limits of Al_2_O_3_ thin films grown using ALD technology by means of strain analysis and pointed out that the critical tensile strain of the film decreases with the increase in the thickness, with the 40 nm alumina film having a lower tensile strain than the 40 nm alumina film, which is the highest tensile strain in the world, where the critical tensile strain of 40 nm Al_2_O_3_ films is 0.95 ± 0.17% and decreases to 0.52 ± 0.22% when the film thickness is increased to 80 nm [112].

**Figure 12:**
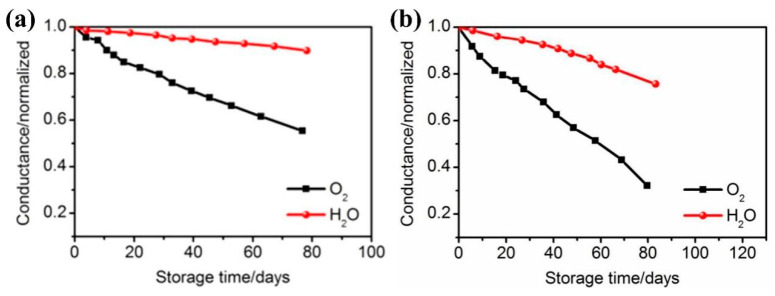
(**a**) Electrical calcium test curves of 50 nm Al_2_O_3_ film. (**b**) Electrical calcium test curves of 50 nm Al_2_O_3_ film after subjected to 1000 cyclic bends with a bending radius of 13 mm (reproduced from [113] with permission from American Chemical Society).

In 2015, Y.-C. Chang’s research team at Feng Chia University used the T-ALD technique to grow Al_2_O_3_ thin films with a thickness of 50 nm at a process temperature of 80 °C by selecting H_2_O and TMA as the oxidant and aluminum source, respectively. Meanwhile, they applied them in the encapsulation of chalcogenide photovoltaic devices and investigated the mechanical stability of the films [113]. As shown in Figure 12, the films have good water vapor and oxygen barrier properties, with WVTR and Oxygen Transmission Rate (OTR) of 9.0 × 10^−4^ g∙m^−2^∙day^−1^ and 1.9 × 10^−3^ cm^3^∙m^−2^∙day^−1^, respectively. However, when the films were bent cyclically at a bending radius of 13 mm for 1000 times, its WVTR and OTR increased to 2.1 × 10^−3^ g∙m^−2^∙day^−1^ and 2.6 × 10^−3^ cm^3^∙m^−2^∙day^−1^, respectively. The films underwent a significant decline in water–oxygen barrier properties after the bending process, indicating that mechanical movement tends to damage the inorganic films and generate additional water–oxygen permeation paths.

In addition, in 2018, a similar phenomenon was found by the research team of K.C. Choi at the Korea Institute of Technology. They found that a 60 nm thick Al_2_O_3_ film grown using the ALD technique yielded a deformation of 0.75% at a bending radius of 1.67 cm, which resulted in an increase in two orders of magnitude in the WVTR [114]. In 2021, the research team of Chen Rong at the Huazhong University of Science and Technology (HUST) investigated the PEN/40 nm Al_2_O_3_ film under cyclic bending for 100 times at bending radii of 7 and 5 mm, respectively. The obvious cracks on the Al_2_O_3_ surface in the SEM images could be found, the film completely failed and lost its water vapor barrier properties [115].

At present, in order to expand the scope of application of inorganic films in the field of flexible encapsulation, organic and inorganic composite structures as well as special flexible structure design are usually used to improve the mechanical properties of films. Organic and inorganic composite materials can combine the unique properties of the two materials: the encapsulation film has not only excellent water vapor barrier properties provided by inorganic materials, but also good mechanical properties provided by organic materials. In addition, the overall mechanical properties of the encapsulation structure can also be improved through some special structural design. For example, improvement could be made to the design of the stacked-film structure to achieve the superposition and neutralization of stress, to the introduction of the defect modification layer to reduce the number of defects within the film to reduce the probability of damage occurring in the defects of the film under mechanical movement, to the adjustment of the neutral surface position to the weak layer of the encapsulation structure to reduce the deformation of the layer in bending, maximizing the suppression of film damage, and to adjusting the position of the neutral plane to the weak layer of the encapsulation structure to reduce the deformation of the layer during bending to minimize film damage.

### 2.2. Organic–Inorganic Composite Package Structure

Organic–inorganic composite encapsulation structure is currently the most commonly used means of flexible thin-film encapsulation, due to the organic material containing a large number of carbon skeletons inside, so that the material itself has excellent mechanical properties [116]. The advantages of inorganic thin film grown using ALD technology lie in its excellent densification, but it also results in the existence of large residual stresses in the film, and, at the same time, the ALD process introduces a defective state inside the inorganic film. In the process of mechanical movement, the defective locations of the film will lead to a concentration of stress in the film, and when the load stress and the residual stress inside the film accumulate too much, the defective locations will preferentially produce a fracture or delamination to release the stress [117], leading to film damage and enhanced water vapor permeability. In contrast, the introduction of organic materials can release film stresses through coupling defects within the ALD inorganic films [32,118]. In addition, due to the excellent ductility of the organic film, it can bring the inorganic film closer to the actual neutral plane position when placed underneath the inorganic film. As a result, the organic–inorganic composite encapsulation structure is able to obtain better mechanical properties while ensuring excellent water vapor barrier properties.

**Figure 13:**
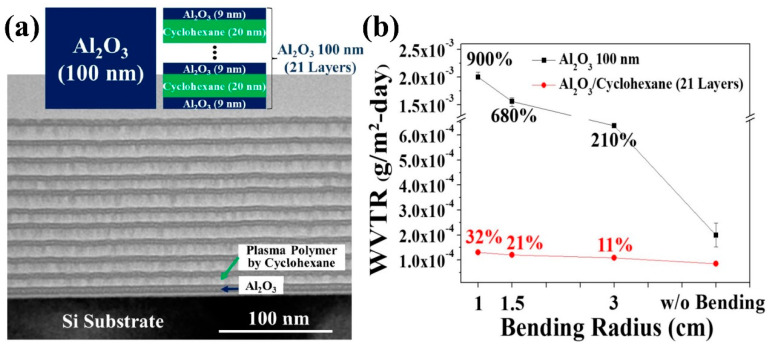
(**a**) Schematic structure of Al_2_O_3_/PP stack with cross-section SEM photographs. (**b**) WVTR before and after bending of films with different structures (reproduced from [119] with permission from American Vacuum Society).

Currently, many research teams have carried out related research around organic–inorganic composite encapsulation structures. In order to improve the mechanical properties of ALD-Al_2_O_3_ thin films, S. J. Kim’s research team at Sungkyunkwan University, South Korea, 2020, introduced plasma polymer (PP) into Al_2_O_3_ thin films in the form of a sandwich structure. The performance of this organic/inorganic laminated structure was analyzed and studied, and the encapsulation structure was finally successfully applied to the encapsulation protection of flexible OLEDs [119]. The inorganic Al_2_O_3_ films were prepared using the ALD process, in which N_2_O plasma and TMA were used as the oxidant and aluminum source, respectively. The organic layers were deposited by plasma polymerization in the same reaction chamber using CHF_3_, benzene or cyclohexane precursors, and the stacked structure through the cyclohexane precursor exhibited better flexibility. A schematic of the Al_2_O_3_/PP stacked layer structure with the WVTR test results of the films before and after bending is shown in Figure 13.

The difference between the two film structures is that the stacked structure is made by dividing 100 nm Al_2_O_3_ into 11 monolayers and inserting 20 nm PP between every two monolayers. The stacked structure exhibits excellent water vapor barrier properties with a WVTR of 8.5 × 10^−5^ g∙m^−2^∙day^−1^, which is 58% lower than that of monolayer Al_2_O_3_, attributed to the ability of the PP layer to act as a water vapor barrier and its coupling effect on the defects within the Al_2_O_3_ layer. For the comparison of the mechanical properties of the two structures, the WVTR of monolayer Al_2_O_3_ increased by 900% after 1000 cycles of bending at a bending radius of 1 cm, whereas the WVTR increment of the organic/inorganic stacked-structure film was only 32% under the same conditions, implying that the organic component enhances the mechanical properties of the film.

In the same year, M. C. Gather’s research team at the University of St. Andrews, UK, fabricated ultrathin flexible OLEDs, with a total thickness of only 12 μm, and was able to maintain its stability after immersion in water. The upper and surfaces of the devices were made of organic and inorganic stacked structural thin films as the high-resistance substrate and encapsulation layer. The inorganic films were 50 nm Al_2_O_3_/ZrO_2_ inorganic nanostacked layers grown using ALD, and the organic films were 3 μm parylene C monolayers grown using CVD. After the encapsulated device was bent 5000 times with a bending radius of 1.5 mm, the device performance did not show any significant degradation [120].

IJP technology is a commonly used organic thin film fabrication technique, which is used in the field of thin-film encapsulation for the coupling of defects in inorganic encapsulated films, and the wrapping coverage of surface-attached particles. In addition, the IJP technology could provide an organic component for flexible encapsulation structures. In 2021, the research team of B.-H.K at ETRI Research Institute in South Korea prepared Al_2_O_3_ monolayers using the PEALD technique for the OLED encapsulation; meanwhile, acrylate polymer films were prepared using IJP technology to form an organic–inorganic stacked structure with the Al_2_O_3_ monolayer. Both the inorganic monolayer and the organic–inorganic stacked structure have excellent water vapor barrier properties, and the WVTRs of both of them are less than 5 × 10^−5^ g∙m^−2^∙day^−1^. However, the stacked structure exhibits better mechanical properties. The WVTR of the Al_2_O_3_/polymer film containing 1.5 cycles of stacking increased to only 2.31 × 10^−4^ g∙m^−2^∙day^−1^ after 10 cycles of bending at a bending radius of 3.2 mm, whereas the WVTR of the Al_2_O_3_ monolayer increased to 4.26 × 10^−1^ g∙m^−2^∙day^−1^ after the same mechanical motions, at which point the film had completely failed [121].

Graphene material is considered a valuable flexible encapsulation material with high permeability, excellent water vapor barrier properties and mechanical properties, and is also often used in organic–inorganic stacked structures. In 2017, H. Kim’s research team at Yonsei University prepared ALD-Al_2_O_3_/CVD-Graphene stacked structures and found that the stacked structures significantly improved in terms of both water vapor barrier properties and mechanical stability compared to the Al_2_O_3_ monolayers [122].

The MLD technology is a monomolecular layer growth process on the substrate surface, and unlike ALD, the MLD process often employs long-chain organic precursors for the growth of organic or organo-inorganic hybridized films. Due to the similarity of the principles of the two processes and the self-limiting nature of the corresponding substrate surface chemistry, respectively, the ALD and MLD technologies are able to be used interchangeably in the same reaction chamber, while the film growth process of both processes is built on top of the substrate surface chemistry; thus, the two films can be combined at the interface in the form of chemisorption to achieve a large adhesion force. This process convenience and the advantage of the adhesion of the stacked structure have made the ALD/MLD technology an important research direction for organic–inorganic stacked encapsulation structures. In 2012, a research team from the University of Colorado, led by S.M. George, proposed that, compared to the ALD-Al_2_O_3_ or MLD-alucone monolayer thin-film structures, the ALD-Al_2_O_3_/MLD-alucone nanostructures can be used for the production of nanocrystalline nanostructures. The nanostacked structures are able to increase the critical tensile strain of the films by enhancing the degree of cross-linking within the films as well as reducing the film brittleness [123]. In addition, the MLD organic films can reduce the residual stresses in the ALD inorganic films by vectorial iteration of stresses or deformation generation to improve their mechanical properties [124,125], and the elastic modulus and hardness of the films in the nanostacked structure are smaller than those of the inorganic film monolayer, which further decrease with the increase in the thickness of the organic layer [126,127].

As the most widely used encapsulation structure, the organic–inorganic composite structure can combine the water vapor barrier properties of inorganic film and the mechanical properties of organic film, so that the advantages and disadvantages of the two films complement each other, showing the value of application in the field of flexible encapsulation. However, although this composite structure well reflects the important role of organic components in flexible encapsulation, the current effect is still difficult to meet the needs of future flexible applications.

**Figure 14:**
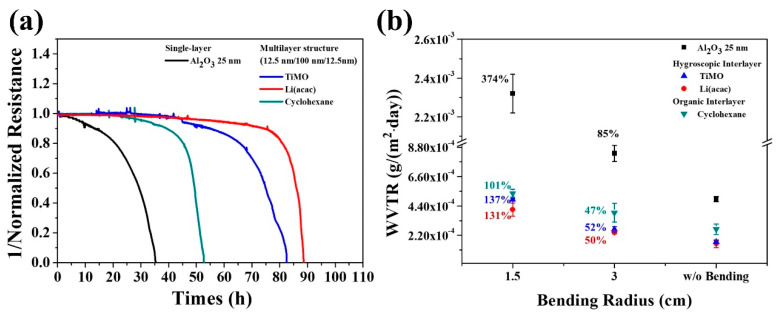
(**a**) Electrical calcium test curves of encapsulated films with inorganic monolayers and organic/inorganic stacked structures. (**b**) Changes in water vapor barrier properties of encapsulated films with inorganic monolayers and organic/inorganic stacked structures before and after bending (reproduced from [128] with permission from Elsevier).

In addition to the enhancement of mechanical properties, the organic components can also serve as desiccants for the encapsulation structure at the same time. In 2019, in order to prolong the lifetime of flexible OLEDs, the research team of Y.J. Choi at Sungkyunkwan University in South Korea chose N_2_O plasma and TMA as the oxidizing agent and the aluminum source, respectively. They prepared 25 nm thick Al_2_O_3_ thin films as the encapsulation layer of the devices by the PEALD technique. Meanwhile, 100 nm TiMO and 100 nm Li(acac) organic films with hygroscopic effect were grown using vacuum thermal evaporation and placed in the middle of Al_2_O_3_ film to form a stacked encapsulation structure [128]. In addition, cyclohexane organic films without hygroscopic effect were added to the comparison experiments to verify the importance of the hygroscopic process, and the results of the film performance tests are shown in Figure 14.

The WVTR of the 25 nm Al_2_O_3_ film exhibits 4.5 × 10^−4^ g∙m^−2^∙day^−1^, which decreases to 2.7 × 10^−4^ g∙m^−2^∙day^−1^ for the stacked encapsulated structure through the introduction of the cyclohexane organic film, which is attributed to the coupling effect of the organic film on the internal defects of the inorganic film to prolong the water vapor penetration path. By replacing the organic layer with TiMO and Li(acac), respectively, the WVTR of the films were further reduced to 1.7 × 10^−4^ g∙m^−2^∙day^−1^ and 1.5 × 10^−4^ g∙m^−2^∙day^−1^, due to the film’s water vapor absorption effect. In addition, the organic/inorganic stacked-film structures were both improved in terms of flexibility compared to the monolayer Al_2_O_3_. After performing 1000 cyclic bends with a bending radius of 1.5 cm, the WVTR increments of the three stacked-structure films were 101%, 137%, and 131%, respectively, which were much smaller than that of the pure inorganic film, which was 374%.

Although the introduction of organic films with hygroscopic effects can simultaneously improve the water vapor barrier properties and mechanical properties of inorganic encapsulation films, this hygroscopic process cannot be adopted as an effective way to optimize the film properties because organic polymer materials tend to expand or even break after absorbing a certain amount of water vapor, resulting in damage to the encapsulation film.

In organic–inorganic composite structures, designing inorganic films as nano-stacked structures can also enhance the mechanical properties by suppressing the defects’ relay. In 2017, K.C. Choi’s research team at the Korea Advanced Institute of Science and Technology (KAIST) compared the performances of Al_2_O_3_ and Al_2_O_3_/ZnO stacks in organic–inorganic composite encapsulant films, respectively, and investigated the defects’ suppression mechanism of the inorganic stacked structures [129].

The inorganic thin films were grown using the T-ALD technique, with TMA, diethylzinc (DEZ) and H_2_O as the aluminum source, zinc source and oxidant, respectively, at a process temperature of 70 °C. In the inorganic stacked structure, ZnO plays a role of defect coupling, effectively inhibiting the occurrence of cracks at the defect location of the Al_2_O_3_ film, and therefore the inorganic stacked structure exhibits excellent mechanical properties. When flexible OLEDs were encapsulated with the two structures and subjected to 1000 cycles of bending with a bending radius of 1 cm, the inorganic stacked-layer encapsulation structure effectively suppressed the generation of cracks, and the devices showed no streak-like corrosion. This work exemplifies the influence of defect states in inorganic thin films on mechanical properties and enhances the mechanical properties of the films by means of defect suppression. Although the inorganic stacked-layer structure was able to reduce the film failure caused by fracture at the defect location, the WVTR of the film increased from 7.87 × 10^−6^ g∙m^−2^∙day^−1^ to 7.78 × 10^−5^ g∙m^−2^∙day^−2^ in 1 cm bending experiments, and this order-of-magnitude degree of decay implies that the mechanical properties are still deficient.

Organic–inorganic composite packages can be introduced inside the inorganic film with organic components in addition to the stacked structure. In 2018, a research team of S.H. Yong from Sungkyunkwan University, South Korea, prepared a carbon-rich Al_2_O_3_ thin-film material by using the PEALD technique, selecting TMA and N_2_O plasma as the aluminum source and oxidant, respectively, and combining it with an inorganic Al_2_O_3_ monolayer as stacked encapsulation. The mechanical properties of the 6.7 nm Al_2_O_3_/2.5 nm 15% C-Al_2_O_3_ film with 2.5 stacking cycles were investigated [130]. In this case, the carbon-rich structure of the Al_2_O_3_ film was able to play a similar role to that of the organic film in the stacked structure. 

Carbon-rich growth of Al_2_O_3_ films is achieved by supplying an excess of TMA precursor during the growth of PEALD-Al_2_O_3_ films, during which the following surface reaction takes place: Al(CH_3_)_3_ + N_2_O → αAl_2_O_3_ + βC_2_H_6_ + ΥN_2_. The WVTR of composite films with 2.5 laminating cycles exhibits a WVTR of 3.3 × 10^−4^ g∙m^−2^∙day^−1^, which is 36% lower compared to the 25 nm Al_2_O_3_ monolayer encapsulated film. In addition, the WVTR increment of the composite film after 1000 cycles of bending at a bending radius of 1.5 cm was only 86%, whereas the WVTR increment of the Al_2_O_3_ monolayer film was as high as 367% after subjecting it to the same mechanical motion.

Although this carbon-rich growth of Al_2_O_3_ can implant organic components into inorganic films and enhance the mechanical properties of the films, this oversaturated supply of precursors introduces a large site resistance during the film growth process, leading to a higher defect density in the film, which creates more water vapor permeation paths and reduces the upper limit of the film’s water vapor barrier properties. 

### 2.3. Flexible Package Structure Design

In addition to organic–inorganic composite packaging structures, there are many thin-film structure design options for flexible applications. For example, stress neutralization is achieved by symmetrically distributing the film on both sides of the neutral plane, mechanical degradation caused by defects in the inorganic film is suppressed by introducing a defect modification layer, and the deformation of the mechanically weak layer is reduced by the modulation of the position of the neutral plane. These structural design solutions are also of great significance in the field of flexible thin-film packaging.

#### 2.3.1. Stress-Neutralizing Structural Design

**Figure 15:**
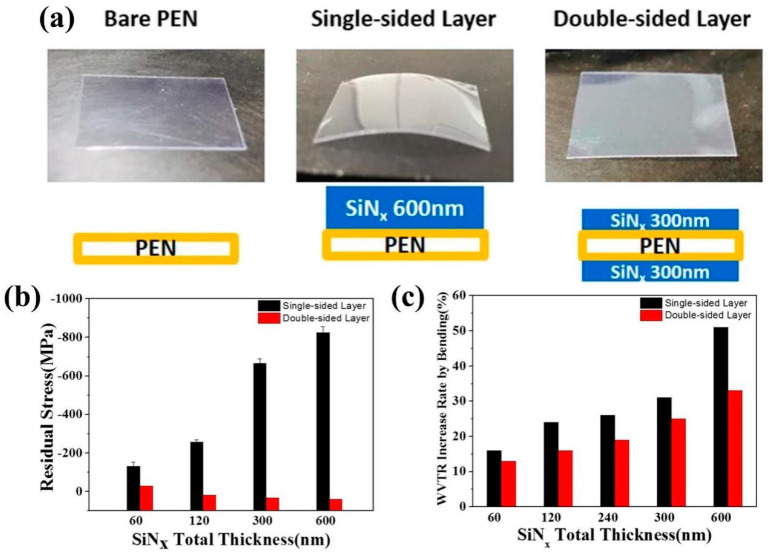
(**a**) Schematic of the unilateral or bilateral growth of the film. (**b**) Comparison of the stresses in the unilateral versus bilateral film. (**c**) Amount of change in the film’s WVTR as a result of bending (reproduced from [131] with permission from AIP Publishing).

Two films with the same stress are grown on both sides of the polymer substrate, and since the two sides have a symmetric stress distribution structure, the substrate does not need to provide additional elastic deformation to match the stress distribution. Thus, the equivalent total stress is zero, and this structural design is considered to be an effective means to be able to enhance the mechanical properties of thin films. In 2018, a research team led by J.S. Park from Sungkyunkwan University, South Korea, grew, in order to alleviate the films’ residual stresses in them, SiN_x_, prepared using PECVD, on one or both sides of the PEN substrate, respectively, and the total thickness of the SiN_x_ films was kept constant in the comparison experiments [131]. The important conclusions from the study are shown in Figure 15. 

It was found that by growing SiN_x_ films on both sides of the PEN substrate bilaterally, the stresses on both sides can cancel each other out, so that the overall structure maintains a lower stress state, and this feature means that the total residual stress of the bilaterally thin-film structure does not increase with the enhancement of the film thickness. In addition, when the total thickness of the SiN_x_ film is 600 nm, the WVTR increment of the bilaterally thin-film structure is 33%, while that of the unilaterally thin-film structure is 51% after cyclic bending for 1000 times at a bending radius of 1.5 cm. Contrastingly, the effect of bending on the bilaterally thin-film structure is smaller than that of the unilaterally thin-film structure in other thicknesses, which proves that the bilaterally thin-film structure can effectively improve the mechanical properties of the film. However, in practical applications, the encapsulated film cannot be distributed symmetrically on both sides of the device. In addition, the biggest role of this structure is to keep the flexible substrate in the high stress film coverage, but it can still maintain the initial flat state to avoid curling. Although the symmetrical distribution of stress can play a total stress neutralization effect, the residual stress inside the single-layer film did not, in fact, weaken; too much residual stress will still limit the mechanical properties of the film.

#### 2.3.2. Repair of Defects in Inorganic Films

Inorganic thin-film materials are often grown using PVD or CVD technology; these growth processes will introduce defective states and residual stresses within the film. In the mechanical movement process of the film, the defective location will lead to film stress concentration. When the load stress and the residual stresses within the film are too large, the defective location will be preferred to produce fracture or delamination to release the stresses, resulting in film damage. This phenomenon leads to further degradation of the mechanical properties of the film. Therefore, the elimination of defects within the film is particularly important.

**Figure 16:**
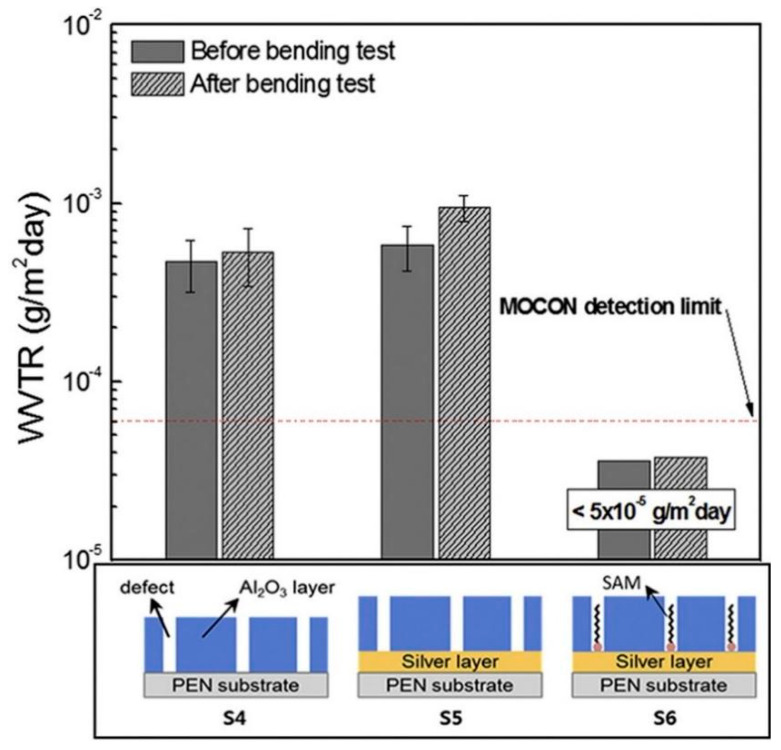
Schematic of different thin-film structures prepared with bending tests (reproduced from [132] with permission from Elsevier).

In 2018, H.G. Kim’s research team at Kyung Hee University in South Korea conducted research on the repair of defects in ALD-Al_2_O_3_ thin films using a self-assembled material, DDT [132], and confirmed that the film defects are also a key factor affecting the mechanical properties of the films, and the results of the thin-film structure and bending tests are shown in Figure 16.

This work deposited an 8 nm thick Ag layer onto the surface of a PEN substrate by thermal evaporation, followed by the deposition of a 40 nm thick Al_2_O_3_ monolayer by LFALD using TMA and O_2_ as the aluminum source and oxidizer, respectively. Self-assembled monolayers were formed using a DDT precursor, which consists of an organosulfur headgroup that interacts with the metal substrate, a methyl endgroup exposed to air, and a 9-carbon alkyl chain between the headgroup and the endgroup. The self-assembled monolayer based on the DDT precursor effectively covered the pinhole defects of the Al_2_O_3_ monolayer. The final multilayer encapsulated structure obtained had a WVTR of less than 5.0 × 10^−5^ g∙m^−2^∙day^−1^ at 38 °C and 100% RH, and it remained unchanged after 25,000 cycles of bending at a 5 mm bending radius. The repair of defects did enhance the mechanical properties of the films, but this defect-filling process via self-assembled materials requires the introduction of additional characterizing materials, such as a metal layer, to allow the self-assembled molecules to undergo directional adsorption, which largely increases the process difficulty. In addition, the size of the self-assembled molecules is sometimes larger than the size of the film defects created by the ALD process, making the defect-repair process using self-assembled molecules even more difficult to carry out.

#### 2.3.3. Neutral Surface Studies

**Figure 17:**
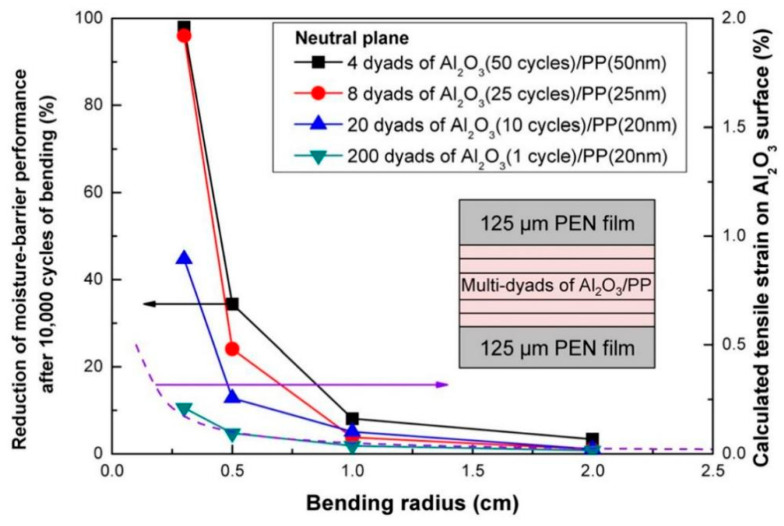
Mechanical properties analysis of the film before and after bending with the encapsulation layer in the neutral plane position (reproduced from [133] with permission from AIP Publishing).

The neutral plane is the plane where the invisible change occurs during the bending process of the film, and the film in the neutral plane position should be well protected. In 2013, the research team of S.-W. Seo at Sungkyunkwan University in South Korea studied the effect of the neutral plane position on the mechanical properties of the film. They prepared an organic/inorganic stacked composite structure, where the organic/inorganic stacked portion in the middle of the two layers of PENs is the traditional flexible encapsulation layer, and the bottom PEN serves as the substrate, and the top PEN serves as the neutral plane regulating layer. The film structure and properties were analyzed as shown in Figure 17 [133].

In this work, an Al_2_O_3_ monolayer was grown using the ALD technique by selecting TMA and H_2_O as the aluminum source and oxidizer, respectively. A PP layer was grown using the PECVD technique by selecting a C6H14 precursor, and the encapsulated film was placed in the neutral plane position through the modulation of the PEN layer. This encapsulation structure displayed a WVTR increment of only 10% after cyclic bending for 10,000 times at a bending radius of 3 mm.

In 2016, Y.C. Han’s research team at the Korea Advanced Institute of Science and Technology (KAIST) prepared S-H nanocomposites/Al_2_O_3_ structures with 3.5 stacking cycles and tuned the neutral plane position by controlling the thickness of the Hybrimer layer [134]. In this work, 3.5 stacking cycles of encapsulated films, including organic–inorganic hybrid nanocomposite monolayers (S-H nanocomposites) and Al_2_O_3_ monolayers, were grown on PET substrates with a thickness of 125 μm. The nanocomposites and the Al_2_O_3_ films were prepared using the solution method and ALD technique, respectively. The neutral plane position of the samples during bending can be changed by regulating the thickness of the uppermost Hybrimer layer, and the intervention of the Hybrimer layer drastically reduces the damage of the encapsulated films during bending, and the WVTR increment generated by 1 cm bending is reduced from 500 times to 2 times. In the final structure, the stacked encapsulant film is placed in the neutral plane position of the sample, and the performance of the encapsulated flexible OLEDs is basically maintained in the initial state after cyclic bending experiments with a bending radius of 1 cm and exposure to 30 °C and 90% RH for 30 days.

Neutral plane regulation is a means to improve the mechanical stability of thin films through structural optimization; however, the design of the neutral plane position needs to take into account all the functional layers of the device, which makes it difficult to design the neutral plane in the face of complex device structures. In addition, while protecting a structure from damage caused by bending by means of neutral plane regulation, the probability of damage to other structures far from the neutral plane position will increase.

In summary, the inorganic encapsulation film could render the superior WVTR, while the existence of strain limits the flexible applications. In contrast, the organic film is suitable for flexible and stretchable application, while the low WVTR could not render efficient protection for devices. Thus, the organic–inorganic hybrid thin-film encapsulation technology could render high WVTR and be suitable for flexible devices, while the multi-layer stacked films significantly enhance the complexity of the fabrication process. Contrastingly, the organic–inorganic hybrid thin-film encapsulation film is still the mainstream encapsulation technology for flexible application. 

## 3. The Development of Flexible Encapsulation Films

The encapsulation technology needs further investigation for application in flexible optoelectronic devices. In 2023, Wang et al. demonstrated that by tailoring the fabrication process of ALD, a homogeneous organic/inorganic hybrid encapsulation could be achieved. By changing the EG/O plasma surface reaction ratio, the component unit of the film could achieve arbitrary ratios. Moreover, the organic/inorganic films were applied in flexible OLED encapsulation, the performance of OLED demonstrated little-to-no chance after bending 10,000 cycles under a 3 mm bending radius [135]. At the same year, the group claimed that by inserting the O plasma into the MLD process, the highly cross-linked, densified flexible encapsulation material AlOC could be fabricated with the WVTR of 1.44 × 10^−5^ g m^−2^ day^−1^. The encapsulated perovskite solar cells could maintain 95% of its initial efficiency after 2400 h under 30℃ and 80% RH [136]. In 2023, Chen et al. demonstrated flexible encapsulation technology based on PECVD. They found that by tuning the ration of N_2_/H_2_ during the fabrication process, the denser, more etch-resistant, higher compressive stress and lower hydrogen content film could be achieved. Moreover, they exhibited an inorganic/organic/inorganic sandwich structure for flexible OLED encapsulation. The encapsulated OLED demonstrated no dark spots under a 2 mm bending radius for 10,000 times [137]. Burger and co-workers thoroughly investigated the encapsulation performance between Si oxide/nitride fabricated by PECVD and metal oxides fabricated by PEALD. Moreover, they combined the inorganic encapsulation film with parylene to form the organic/inorganic hybrid encapsulation film, and they found that the parylene-AlO_x_ demonstrated the most effective solutions. WVTR could reach up to 3.1× 10^−4^ g m^−2^ day^−1^ under 38℃ and 90% RH [138]. Choi et al. achieved the foldable and washable textile-based OLEDs based on the pV3D3 and Al_2_O_3_/TiO_2_ bilayer encapsulation structure. The Al_2_O_3_ and TiO_2_ were deposited by ALD at near-room-temperature, which was beneficial for the OLED devices. Combined with the CVD-based pV3D3 polymer, the flexibility and waterproof property was significantly enhanced, the wearable OLED could emit red light even folded under water during hand-washing [139], and foldable and washable textile-based OLEDs possessed a multi-functional near-room-temperature encapsulation layer for smart e-textiles. Chen et al. also demonstrated the advantages of the organic/inorganic hybrid encapsulation layer. They utilized the PDMS as the organic sublayers and utilized O_2_ plasma to provide the nucleation sites for the deposition of the Al_2_O_3_ layer. Furthermore, the epoxy layer was used to improve the strain condition under the bending test. Finally, the hybrid encapsulation demonstrated superior isolation and mechanical reliability, and the lifetime of blue OLED could reach up to 370 h under 25 °C and 60% RH [115], Flexible PDMS/Al2O3 Nanolaminates for the Encapsulation of Blue OLEDs.

## 4. Summary and Outlook

Flexible OLEDs have many advantages, such as thinness, lightness, shock resistance, etc., which can provide more possibilities for the diversity of product forms, and they have potential application value in display, lighting, and medical industries. In recent years, domestic and foreign conferences on the theme of “wearable optoelectronics” have been favored. OLEDs, ranging from the rigid form to the curved form, are now foldable, in order to meet the increasing demand for flexibility and the minimum bending radius of the product to withstand the millimeter level, which is a great test for the thin-film encapsulation technology. This is a great test for thin-film packaging technology.

Existing thin-film encapsulation technologies are mainly based on traditional thin-film fabrication means, such as CVD, IJP, sputtering, spin-coating and thermal evaporation. Although inorganic thin-film materials have good water vapor barrier properties, their poor mechanical properties have also been a key issue limiting their application in flexible packaging, such as low film ductility, low fracture toughness, high brittleness, etc. Mechanical stress or deformation of the film will limit the durability and reliability of the film in the process. Organic materials are flexible, but due to the process characteristics of the preparation method, organic films contain more defects that provide environmental water vapor penetration paths, making their barrier properties unable to meet the demands of optoelectronic devices. The method of organic/inorganic stacking can combine the advantages of inorganic and organic materials, using the excellent barrier properties of inorganic materials to form a good barrier to water vapor permeation, and the organic layer can effectively relieve the film stress, separating the neutral surface, which brings good mechanical properties. However, the method requires a thicker organic film coupled with an inorganic film, which keeps the inorganic film position away from the neutral surface position. The deformation of the outer surface of the encapsulated film would increase during device bending, introducing higher additional stresses. The probability of film damage is thus elevated, lowering the upper limit of the mechanical properties. Moreover, the adhesion strength at the organic/inorganic interface is low and prone to delamination, which will be reflected in the strict mechanical motion process. Therefore, the organic/inorganic stacked layer structure can no longer meet the future needs of flexible device protection. Although placing the encapsulation layer in the neutral position can effectively protect it from mechanical movement damage, this method is only a compromise program. In the face of complex device structure, the neutral surface design difficulty is also higher.

Therefore, the authors believe that for the future development of flexible optoelectronic devices, including flexible OLEDs, the existing high level of thin-film fabrication and encapsulation technology can no longer satisfy, and research can no longer be limited by traditional process methods and empirical structures. The development of new processes, materials and structures is the only way to realize the future standard of flexible packaging.

## References

[1] Hong G., Gan X., Leonhardt C., Zhang Z., Seibert J., Busch J.M., Braese S. (2021). A Brief History of OLEDs-Emitter Development and Industry Milestones. Adv. Mater..

[2] Geffroy B., le Roy P., Prat C. (2006). Organic light-emitting diode (OLED) technology: Materials, devices and display technologies. Polym. Int..

[3] Kulkarni A.P., Tonzola C.J., Babel A., Jenekhe S.A. (2004). Electron transport materials for organic light-emitting diodes. Chem. Mater..

[4] Uoyama H., Goushi K., Shizu K., Nomura H., Adachi C. (2012). Highly efficient organic light-emitting diodes from delayed fluorescence. Nature.

[5] Sun Y.R., Giebink N.C., Kanno H., Ma B.W., Thompson M.E., Forrest S.R. (2006). Management of singlet and triplet excitons for efficient white organic light-emitting devices. Nature.

[6] Li S., Wang L., Tang D., Cho Y., Liu X., Zhou X., Lu L., Zhang L., Takeda T., Hirosaki N. (2018). Achieving High Quantum Efficiency Narrow-Band beta-Sialon:Eu^2+^ Phosphors for High-Brightness LCD Backlights by Reducing the Eu^3+^ Luminescence Killer. Chem. Mater..

[7] Chiu H.-J., Cheng S.-J. (2007). LED backlight driving system for large-scale LCD panels. IEEE Trans. Ind. Electron..

[8] Park C.H., Kim J.G., Jung S.-G., Lee D.J., Park Y.W., Ju B.-K. (2019). Optical characteristics of refractive- index-matching diffusion layer in organic light-emitting diodes. Sci. Rep..

[9] Choi Y., Oh S.-W., Choi T.-H., Yu B.-H., Yoon T.-H. (2019). Formation of polymer structure by thermally-induced phase separation for a dye-doped liquid crystal light shutter. Dye. Pigment..

[10] Ma H., Yip H.-L., Huang F., Jen A.K.Y. (2010). Interface Engineering for Organic Electronics. Adv. Funct. Mater..

[11] Gross M., Muller D.C., Nothofer H.G., Scherf U., Neher D., Brauchle C., Meerholz K. (2000). Improving the performance of doped pi-conjugated polymers for use in organic light-emitting diodes. Nature.

[12] Wang S., Zhang H., Zhang B., Xie Z., Wong W.-Y. (2020). Towards high-power-efficiency solution-processed OLEDs: Material and device perspectives. Mater. Sci. Eng. R-Rep..

[13] Li C.-C., Tseng H.-Y., Liao H.-C., Chen H.-M., Hsieh T., Lin S.-A., Jau H.-C., Wu Y.-C., Hsu Y.-L., Hsu W.-H. (2017). Enhanced image quality of OLED transparent display by cholesteric liquid crystal back-panel. Opt. Express.

[14] Vashishtha P., Ng M., Shivarudraiah S.B., Halpert J.E. (2019). High Efficiency Blue and Green Light-Emitting Diodes Using Ruddlesden-Popper Inorganic Mixed Halide Perovskites with Butylammonium Interlayers. Chem. Mater..

[15] Zou S.-J., Shen Y., Xie F.-M., Chen J.-D., Li Y.-Q., Tang J.-X. (2020). Recent advances in organic light-emitting diodes: Toward smart lighting and displays. Mater. Chem. Front..

[16] Bhagat S.A., Borghate S.V., Kalyani N.T., Dhoble S.J. (2015). Novel Na+ doped Alq(3) hybrid materials for organic light-emitting diode (OLED) devices and flat panel displays. Luminescence.

[17] Lian C., Piksa M., Yoshida K., Persheyev S., Pawlik K.J., Matczyszyn K., Samuel I.D.W. (2019). Flexible organic light-emitting diodes for antimicrobial photodynamic therapy. NPJ Flex. Electron..

[18] Reineke S., Lindner F., Schwartz G., Seidler N., Walzer K., Luessem B., Leo K. (2009). White organic light-emitting diodes with fluorescent tube efficiency. Nature.

[19] White M.S., Kaltenbrunner M., Glowacki E.D., Gutnichenko K., Kettlgruber G., Graz I., Aazou S., Ulbricht C., Egbe D.A.M., Miron M.C. (2013). Ultrathin, highly flexible and stretchable PLEDs. Nat. Photonics.

[20] Xu R.-P., Li Y.-Q., Tang J.-X. (2016). Recent advances in flexible organic light-emitting diodes. J. Mater. Chem. C.

[21] Liu Y.-F., Feng J., Bi Y.-G., Yin D., Sun H.-B. (2019). Recent Developments in Flexible Organic Light-Emitting Devices. Adv. Mater. Technol..

[22] Popovic Z.D., Aziz H. (2002). Reliability and degradation of small molecule-based organic light-emitting devices (OLEDs). IEEE J. Sel. Top. Quantum Electron..

[23] Azrain M., Omar G., Mansor M., Fadzullah S., Lim L. (2019). Failure mechanism of organic light emitting diodes (OLEDs) induced by hygrothermal effect. Opt. Mater..

[24] Swayamprabha S.S., Dubey D.K., Shahnawaz, Yadav R.A.K., Nagar M.R., Sharma A., Tung F.-C., Jou J.-H. (2021). Approaches for Long Lifetime Organic Light Emitting Diodes. Adv. Sci..

[25] Fukagawa H. (2018). Molecular Design and Device Design to Improve Stabilities of Organic Light-Emitting Diodes. J. Photopolym. Sci. Technol..

[26] Ghosh A.P., Gerenser L.J., Jarman C.M., Fornalik J.E. (2005). Thin-film encapsulation of organic light-emitting devices. Appl. Phys. Lett..

[27] Wang R., Mujahid M., Duan Y., Wang Z.-K., Xue J., Yang Y. (2019). A Review of Perovskites Solar Cell Stability. Adv. Funct. Mater..

[28] Jeong E.G., Kwon J.H., Kang K.S., Jeong S.Y., Choi K.C. (2020). A review of highly reliable flexible encapsulation technologies towards rollable and foldable OLEDs. J. Inf. Disp..

[29] Park S.H.K., Oh J., Hwang C.S., Lee J.I., Yang Y.S., Chu H.Y. (2005). Ultrathin film encapsulation of an OLED by ALD. Electrochem. Solid State Lett..

[30] Moro L., Krajewski T.A., Rutherford N.M., Philips O., Visser R.J., Gross M.E., Bennett W.D., Graff G.L. Process and design of a multilayer thin film encapsulation of passive matrix OLED displays. Proceedings of the Conference on Organic Light-Emitting Materials and Devices VII.

[31] Wu J., Fei F., Wei C., Chen X., Nie S., Zhang D., Su W., Cui Z. (2018). Efficient multi-barrier thin film encapsulation of OLED using alternating Al2O3 and polymer layers. RSC Adv..

[32] Lee S., Han J.H., Lee S.H., Baek G.H., Park J.S. (2019). Review of Organic/Inorganic Thin Film Encapsulation by Atomic Layer Deposition for a Flexible OLED Display. JOM.

[33] Madogni V.I., Agbomahéna M., Kounouhéwa B.B., Douhéret O., Lazzaroni R. (2018). Effects of Residual Oxygen in the Degradation of the Performance of Organic Bulk Heterojunction Solar Cells: Stability, Role of the Encapsulation. Adv. Mater. Phys. Chem..

[34] Park M.-H., Han T.-H., Kim Y.-H., Jeong S.-H., Lee Y., Seo H.-K., Cho H., Lee T.-W. (2015). Flexible organic light-emitting diodes for solid-state lighting. J. Photonics Energy.

[35] Zhang D., Huang T., Duan L. (2020). Emerging Self-Emissive Technologies for Flexible Displays. Adv. Mater..

[36] Gu G., Burrows P.E., Venkatesh S., Forrest S.R., Thompson M.E. (1997). Vacuum-deposited, nonpolymeric flexible organic light-emitting devices. Opt. Lett..

[37] Ai X., Evans E.W., Dong S., Gillett A.J., Guo H., Chen Y., Hele T.J.H., Friend R.H., Li F. (2018). Efficient radical-based light-emitting diodes with doublet emission. Nature.

[38] Lu Q., Yang Z.C., Meng X., Yue Y.F., Ahmad M.A., Zhang W.J., Zhang S.S., Zhang Y.Q., Liu Z.H., Chen W. (2021). A Review on Encapsulation Technology from Organic Light Emitting Diodes to Organic and Perovskite Solar Cells. Adv. Funct. Mater..

[39] Chwang A.B., Rothman M.A., Mao S.Y., Hewitt R.H., Weaver M.S., Silvernail J.A., Rajan K., Hack M., Brown J.J., Chu X. (2003). Thin film encapsulated flexible organic electroluminescent displays. Appl. Phys. Lett..

[40] Park J.S., Chae H., Chung H.K., Lee S.I. (2011). Thin film encapsulation for flexible AM-OLED: A review. Semicond. Sci. Technol..

[41] Kwon S.-K., Baek J.-H., Choi H.-C., Kim S.K., Lampande R., Pode R., Kwon J.H. (2019). Degradation of OLED performance by exposure to UV irradiation. RSC Adv..

[42] Lee Y.I., Jeon N.J., Kim B.J., Shim H., Yang T.Y., Seok S.I., Seo J., Im S.G. (2018). A low-temperature thin-film encapsulation for enhanced stability of a highly efficient perovskite solar cell. Adv. Energy Mater..

[43] Choi J.-H., Ha M.-J., Park J.C., Park T.J., Kim W.-H., Lee M.-J., Ahn J.-H. (2022). A Strategy for Wafer-Scale Crystalline MoS_2_ Thin Films with Controlled Morphology Using Pulsed Metal-Organic Chemical Vapor Deposition at Low Temperature. Adv. Mater. Interfaces.

[44] Salameh F., Al Haddad A., Picot A., Canale L., Zissis G., Chabert M., Maussion P. (2019). Modeling the Luminance Degradation of OLEDs Using Design of Experiments. IEEE Trans. Ind. Appl..

[45] Batey J., Tierney E. (1986). Low-temperature deposition of high-quality silicon dioxide by plasma-enhanced chemical vapor deposition. J. Appl. Phys..

[46] Kim J., Hwang J.H., Kwon Y.W., Bae H.W., An M., Lee W., Lee D. (2021). Hydrogen-assisted low-temperature plasma-enhanced chemical vapor deposition of thin film encapsulation layers for top-emission organic light-emitting diodes. Org. Electron..

[47] Zikulnig J., Chang S., Bito J., Rauter L., Roshanghias A., Carrara S., Kosel J. (2023). Printed Electronics Technologies for Additive Manufacturing of Hybrid Electronic Sensor Systems. Adv. Sens. Res..

[48] Derby B. (2010). Inkjet Printing of Functional and Structural Materials: Fluid Property Requirements, Feature Stability, and Resolution. Annu. Rev. Mater. Res..

[49] Wang T., Sun T., Xie C., Wang Y., Qin C., Zhang Z., Zhou W., Zhang S. (2019). SID Symposium Digest of Technical Papers.

[50] Crowell J.E. (2003). Chemical methods of thin film deposition: Chemical vapor deposition, atomic layer deposition, and related technologies. J. Vac. Sci. Technol. A Vac. Surf. Film..

[51] Sabzi M., Anijdan S.H.M., Shamsodin M., Farzam M., Hojjati-Najafabadi A., Feng P., Park N., Lee U. (2023). A Review on Sustainable Manufacturing of Ceramic-Based Thin Films by Chemical Vapor Deposition (CVD): Reactions Kinetics and the Deposition Mechanisms. Coatings.

[52] Puurunen R.L. (2005). Surface chemistry of atomic layer deposition: A case study for the trimethylaluminum/water process. J. Appl. Phys..

[53] George S., Ott A., Klaus J. (1996). Surface chemistry for atomic layer growth. J. Phys. Chem..

[54] Forte M.A., Silva R.M., Tavares C.J., Silva R.F.E. (2021). Is Poly(methyl methacrylate) (PMMA) a Suitable Substrate for ALD? A Review. Polymers.

[55] Kovacs R.L., Csontos M., Gyongyosi S., Elek J., Parditka B., Deak G., Kuki A., Keki S., Erdelyi Z. (2021). Surface characterization of plasma-modified low density polyethylene by attenuated total reflectance fourier-transform infrared (ATR-FTIR) spectroscopy combined with chemometrics. Polym. Test..

[56] Clark M.P., Muneshwar T., Xiong M., Cadien K., Ivey D.G. (2019). Saturation Behavior of Atomic Layer Deposition MnOx from Bis(Ethylcyclopentadienyl) Manganese and Water: Saturation Effect on Coverage of Porous Oxygen Reduction Electrodes for Metal-Air Batteries. ACS Appl. Nano Mater..

[57] George S.M. (2010). Atomic Layer Deposition: An Overview. Chem. Rev..

[58] de la Huerta C.M., Huong N.V., Dedulle J.-M., Bellet D., Jimenez C., Munoz-Rojas D. (2019). Influence of the Geometric Parameters on the Deposition Mode in Spatial Atomic Layer Deposition: A Novel Approach to Area-Selective Deposition. Coatings.

[59] Shahmohammadi M., Mukherjee R., Takoudis C.G., Diwekar U.M. (2021). Optimal design of novel precursor materials for the atomic layer deposition using computer-aided molecular design. Chem. Eng. Sci..

[60] Fabreguette F.H., Wind R.A., George S.M. (2006). Ultrahigh x-ray reflectivity from W/ Al^2^O^3^ multilayers fabricated using atomic layer deposition. Appl. Phys. Lett..

[61] Wu Y., Yang X., Chen H., Zhang K., Qin C., Liu J., Peng W., Islam A., Bi E., Ye F. (2014). Highly compact TiO2 layer for efficient hole-blocking in perovskite solar cells. Appl. Phys. Express.

[62] Graniel O., Weber M., Balme S., Miele P., Bechelany M. (2018). Atomic layer deposition for biosensing applications. Biosens. Bioelectron..

[63] Yu Y., Zhang Z., Yin X., Kvit A., Liao Q., Kang Z., Yan X., Zhang Y., Wang X. (2017). Enhanced photoelectrochemical effciency and stability using a conformal TiO2 film on a black silicon photoanode. Nat. Energy.

[64] Sheng J., Lee J.-H., Choi W.-H., Hong T., Kim M., Park J.-S. (2018). Review Article: Atomic layer deposition for oxide semiconductor thin film transistors: Advances in research and development. J. Vac. Sci. Technol. A.

[65] Song E., Lee Y.K., Li R., Li J., Jin X., Yu K.J., Xie Z., Fang H., Zhong Y., Du H. (2018). Transferred, Ultrathin Oxide Bilayers as Biofluid Barriers for Flexible Electronic Implants. Adv. Funct. Mater..

[66] Sheng J., Hong T., Kang D., Yi Y., Lim J.H., Park J.-S. (2019). Design of InZnSnO Semiconductor Alloys Synthesized by Supercycle Atomic Layer Deposition and Their Rollable Applications. ACS Appl. Mater. Interfaces.

[67] Multia J., Heiska J., Khayyami A., Karppinen M. (2020). Electrochemically Active In Situ Crystalline Lithium-Organic Thin Films by ALD/MLD. ACS Appl. Mater. Interfaces.

[68] Ponja S.D., Williamson B.A.D., Sathasivam S., Scanlon D.O., Parkin I.P., Carmalt C.J. (2018). Enhanced electrical properties of antimony doped tin oxide thin films deposited via aerosol assisted chemical vapour deposition. J. Mater. Chem. C.

[69] Islam M.R., Rahman M., Farhad S.F.U., Podder J. (2019). Structural, optical and photocatalysis properties of sol-gel deposited Al-doped ZnO thin films. Surf. Interfaces.

[70] International Technology Roadmap for Semiconductors. 2007 Edition. http://www.itrs.net/.

[71] Chou C.-T., Yu P.-W., Tseng M.-H., Hsu C.-C., Shyue J.-J., Wang C.-C., Tsai F.-Y. (2013). Transparent Conductive Gas-Permeation Barriers on Plastics by Atomic Layer Deposition. Adv. Mater..

[72] Li Y., Xiong Y., Yang H., Cao K., Chen R. (2020). Thin film encapsulation for the organic light-emitting diodes display via atomic layer deposition. J. Mater. Res..

[73] Johnson R.W., Hultqvist A., Bent S.F. (2014). A brief review of atomic layer deposition: From fundamentals to applications. Mater. Today.

[74] Kim L.H., Jang J.H., Jeong Y.J., Kim K., Baek Y., Kwon H.-J., An T.K., Nam S., Kim S.H., Jang J. (2017). Highly-impermeable Al_2_O_3_/HfO_2_ moisture barrier films grown by low-temperature plasma-enhanced atomic layer deposition. Org. Electron..

[75] Zhu Z., Merdes S., Ylivaara O.M.E., Mizohata K., Heikkila M.J., Savin H. (2020). Al_2_O_3_ Thin Films Prepared by a Combined Thermal-Plasma Atomic Layer Deposition Process at Low Temperature for Encapsulation Applications. Phys. Status Solidi A-Appl. Mater. Sci..

[76] Oh J., Shin S., Park J., Ham G., Jeon H. (2016). Characteristics of Al_2_O_3_/ZrO_2_ laminated films deposited by ozone-based atomic layer deposition for organic device encapsulation. Thin Solid Film..

[77] Li C., Cauwe M., Yang Y., Schaubroeck D., Mader L., de Beeck M.O. (2019). Ultra-Long-Term Reliable Encapsulation Using an Atomic Layer Deposited HfO_2_/Al_2_O_3_/HfO_2_ Triple-Interlayer for Biomedical Implants. Coatings.

[78] Lee Y., Seo S., Oh I.-K., Lee S., Kim H. (2019). Effects of O_2_ plasma treatment on moisture barrier properties of SiO_2_ grown by plasma-enhanced atomic layer deposition. Ceram. Int..

[79] Lee U.S., Choi J.S., Yang B.S., Oh S., Kim Y.J., Oh M.S., Heo J., Kim H.J. (2013). Formation of a Bilayer of ALD-SiO_2_ and Sputtered Al_2_O_3_/ZrO_2_ Films on Polyethylene Terephthalate Substrates as a Moisture Barrier. Ecs Solid State Lett..

[80] Kukli K., Kemell M., Castan H., Duenas S., Seemen H., Rahn M., Link J., Stern R., Heikkila M.J., Ritala M. (2018). Atomic Layer Deposition and Performance of ZrO_2_-Al_2_O_3_ Thin Films. ECS J. Solid State Sci. Technol..

[81] Yu D., Yang Y.-Q., Chen Z., Tao Y., Liu Y.-F. (2016). Recent progress on thin-film encapsulation technologies for organic electronic devices. Opt. Commun..

[82] Yun S.J., Lim J.W., Lee J.H. (2004). Low-temperature deposition of aluminum oxide on polyethersulfone substrate using plasma-enhanced atomic layer deposition. Electrochem. Solid State Lett..

[83] Yang Y.-Q., Duan Y., Chen P., Sun F.-B., Duan Y.-H., Wang X., Yang D. (2013). Realization of Thin Film Encapsulation by Atomic Layer Deposition of Al2O3 at Low Temperature. J. Phys. Chem. C.

[84] Lim J.W., Yun S.J. (2004). Electrical properties of alumina films by plasma-enhanced atomic layer deposition. Electrochem. Solid State Lett..

[85] Iliescu C., Avram M., Chen B., Popescu A., Dumitrescu V., Poenar D.P., Sterian A., Vrtacnik D., Amon S., Sterian P. (2011). Residual stress in thin films PECVD depositions: A review. J. Optoelectron. Adv. Mater..

[86] Zhang S., Shi W., Siegler T.D., Gao X., Ge F., Korgel B.A., He Y., Li S., Wang X. (2019). An All-Inorganic Colloidal Nanocrystal Flexible Polarizer. Angew. Chem. Int. Ed..

[87] Yuan Y., Xie S., Ding C., Shi X., Xu J., Li K., Zhao W. (2020). Fabricating flexible wafer-size inorganic semiconductor devices. J. Mater. Chem. C.

[88] Yeom B., Kim S., Cho J., Hahn J., Char K. (2006). Effect of interfacial adhesion on the mechanical properties of organic/inorganic hybrid nanolaminates. J. Adhes..

[89] Yeom B., Jeong A., Lee J., Char K. (2016). Enhancement of fracture toughness in organic/inorganic hybrid nanolaminates with ultrathin adhesive layers. Polymer.

[90] Tu N., Jiang J., Chen Q., Liao J., Liu W., Yang Q., Jiang L., Zhou Y. (2019). Flexible ferroelectric capacitors based on Bi3.15Nd0.85Ti3O12/muscovite structure. Smart Mater. Struct..

[91] Niu R., Liu G., Ding X., Sun J. (2008). Ductility of metal thin films in flexible electronics. Sci. China Ser. E-Technol. Sci..

[92] Zhao Y., Zhang L., Liu J., Adair K., Zhao F., Sun Y., Wu T., Bi X., Amine K., Lu J. (2021). Atomic/molecular layer deposition for energy storage and conversion. Chem. Soc. Rev..

[93] Multia J., Karppinen M. (2022). Atomic/Molecular Layer Deposition for Designer’s Functional Metal-Organic Materials. Adv. Mater. Interfaces.

[94] McIntee O.M., Welch B.C., Greenberg A.R., George S.M., Bright V.M. (2022). Elastic modulus of polyamide thin films formed by molecular layer deposition. Polymer.

[95] Lee B.H., Lee K.H., Im S., Sung M.M. (2009). Vapor-Phase Molecular Layer Deposition of Self-Assembled Multilayers for Organic Thin-Film Transistor. J. Nanosci. Nanotechnol..

[96] Muneshwar T., Cadien K. (2018). Surface reaction kinetics in atomic layer deposition: An analytical model and experiments. J. Appl. Phys..

[97] Wang H., Wang Z., Xu X., Liu Y., Chen C., Chen P., Hu W., Duan Y. (2019). Multiple short pulse process for low-temperature atomic layer deposition and its transient steric hindrance. Appl. Phys. Lett..

[98] Schaepkens M., Kim T.W., Erlat A.G., Yan M., Flanagan K.W., Heller C.M., McConnelee P.A. (2004). Ultrahigh barrier coating deposition on polycarbonate substrates. J. Vac. Sci. Technol. A.

[99] Perrotta A., Aresta G., van Beekum E.R.J., Palmans J., van de Weijer P., van de Sanden M.C.M.R., Kessels W.M.M.E., Creatore M. (2015). The impact of the nano-pore filling on the performance of organosilicon-based moisture barriers. Thin Solid Film..

[100] Chen D.H., Ozaki S. (2009). Stress concentration due to defects in a honeycomb structure. Compos. Struct..

[101] Gao Y., Chen Y. (2019). Sawing stress of SiC single crystal with void defect in diamond wire saw slicing. Int. J. Adv. Manuf. Technol..

[102] Carcia P.F., McLean R., Reilly M., Groner M., George S. (2006). Ca test of Al_2_O_3_ gas diffusion barriers grown by atomic layer deposition on polymers. Appl. Phys. Lett..

[103] Choi H., Shin S., Jeon H., Choi Y., Kim J., Kim S., Chung S.C., Oh K. (2016). Fast spatial atomic layer deposition of Al_2_O_3_ at low temperature (<100 degrees C) as a gas permeation barrier for flexible organic light-emitting diode displays. J. Vac. Sci. Technol. A.

[104] Keuning W., van de Weijer P., Lifka H., Kessels W.M.M., Creatore M. (2012). Cathode encapsulation of organic light emitting diodes by atomic layer deposited Al_2_O_3_ films and Al_2_O_3_/a-SiNx:H stacks. J. Vac. Sci. Technol. A.

[105] Meyer J., Görrn P., Bertram F., Hamwi S., Winkler T., Johannes H.H., Weimann T., Hinze P., Riedl T., Kowalsky W. (2009). Al_2_O_3_/ZrO_2_ nanolaminates as ultrahigh gas-diffusion barriers—A strategy for reliable encapsulation of organic electronics. Adv. Mater..

[106] Seo S.-W., Jung E., Chae H., Cho S.M. (2012). Optimization of Al_2_O_3_/ZrO_2_ nanolaminate structure for thin-film encapsulation of OLEDs. Org. Electron..

[107] Kim L.H., Kim K., Park S., Jeong Y.J., Kim H., Chung D.S., Kim S.H., Park C.E. (2014). Al_2_O_3_/TiO_2_ nanolaminate thin film encapsulation for organic thin film transistors via plasma-enhanced atomic layer deposition. Acs Appl. Mater. Interfaces.

[108] Choi H., Lee S., Jung H., Shin S., Ham G., Seo H., Jeon H. (2013). Moisture Barrier Properties of Al_2_O_3_ Films deposited by Remote Plasma Atomic Layer Deposition at Low Temperatures. Jpn. J. Appl. Phys..

[109] Pilz J., Perrotta A., Leising G., Coclite A.M. (2020). ZnO Thin Films Grown by Plasma-Enhanced Atomic Layer Deposition: Material Properties Within and Outside the “Atomic Layer Deposition Window”. Phys. Status Solidi A-Appl. Mater. Sci..

[110] Jang W., Jeon H., Kang C., Song H., Park J., Kim H., Seo H., Leskela M., Jeon H. (2014). Temperature dependence of silicon nitride deposited by remote plasma atomic layer deposition. Phys. Status Solidi A-Appl. Mater. Sci..

[111] Wang H., Zhao Y., Wang Z., Liu Y., Zhao Z., Xu G., Han T.-H., Lee J.-W., Chen C., Bao D. (2020). Hermetic seal for perovskite solar cells: An improved plasma enhanced atomic layer deposition encapsulation. Nano Energy.

[112] Jen S.-H., Bertrand J.A., George S.M. (2011). Critical tensile and compressive strains for cracking of Al_2_O_3_ films grown by atomic layer deposition. J. Appl. Phys..

[113] Chang C.-Y., Lee K.-T., Huang W.-K., Siao H.-Y., Chang Y.-C. (2015). High-Performance, Air-Stable, Low-Temperature Processed Semitransparent Perovskite Solar Cells Enabled by Atomic Layer Deposition. Chem. Mater..

[114] Kwon J.H., Jeong E.G., Jeon Y., Kim D.-G., Lee S., Choi K.C. (2018). Design of highly water resistant, impermeable, and flexible thin-film encapsulation based on inorganic/organic hybrid layers. Acs Appl. Mater. Interfaces.

[115] Li Y., Xiong Y., Cao W., Zhu Q., Lin Y., Zhang Y., Liu M., Yang F., Cao K., Chen R. (2021). Flexible PDMS/Al_2_O_3_ nanolaminates for the encapsulation of blue OLEDs. Adv. Mater. Interfaces.

[116] Casillas G., Mayoral A., Liu M., Ponce A., Artyukhov V.I., Yakobson B.I., Jose-Yacaman M. (2014). New insights into the properties and interactions of carbon chains as revealed by HRTEM and DFT analysis. Carbon.

[117] Shugurov A.R., Panin A.V. Mechanisms of stress generation and relaxation in thin films and coatings. Proceedings of the International Conference on Physical Mesomechanics of Multilevel Systems 2014.

[118] Li Y.-S., Tsai C.-H., Kao S.-H., Wu I.W., Chen J.-Z., Wu C.-I., Lin C.-F., Cheng I.C. (2013). Single-layer organic-inorganic-hybrid thin-film encapsulation for organic solar cells. J. Phys. D-Appl. Phys..

[119] Kim S.J., Yong S.H., Choi Y.J., Hwangbo H., Yang W.-Y., Chae H. (2020). Flexible Al_2_O_3_/plasma polymer multilayer moisture barrier films deposited by a spatial atomic layer deposition process. J. Vac. Sci. Technol. A.

[120] Keum C., Murawski C., Archer E., Kwon S., Mischok A., Gather M.C. (2020). A substrateless, flexible, and water-resistant organic light-emitting diode. Nat. Commun..

[121] Kwon B.-H., Joo C.W., Cho H., Kang C.-M., Yang J.-H., Shin J.-W., Kim G.H., Choi S., Nam S., Kim K. (2021). Organic/Inorganic Hybrid Thin-Film Encapsulation Using Inkjet Printing and PEALD for Industrial Large-Area Process Suitability and Flexible OLED Application. Acs Appl. Mater. Interfaces.

[122] Nam T., Park Y.J., Lee H., Oh I.-K., Ahn J.-H., Cho S.M., Kim H. (2017). A composite layer of atomic-layer-deposited Al_2_O_3_ and graphene for flexible moisture barrier. Carbon.

[123] Jen S.-H., Lee B.H., George S.M., McLean R.S., Carcia P.F. (2012). Critical tensile strain and water vapor transmission rate for nanolaminate films grown using Al_2_O_3_ atomic layer deposition and alucone molecular layer deposition. Appl. Phys. Lett..

[124] Chen G., Weng Y., Sun F., Zhou X., Wu C., Yan Q., Guo T., Zhang Y. (2019). Low-temperature atomic layer deposition of Al_2_O_3_/alucone nanolaminates for OLED encapsulation. RSC Adv..

[125] Chen T., Wuu D., Wu C., Chiang C., Chen Y., Horng R.-H. (2006). High-performance transparent barrier films of SiO_x_/SiN_x_ stacks on flexible polymer substrates. J. Electrochem. Soc..

[126] Leterrier Y. (2003). Durability of nanosized oxygen-barrier coatings on polymers. Prog. Mater. Sci..

[127] Lewis J.S., Weaver M.S. (2004). Thin-film permeation-barrier technology for flexible organic light-emitting devices. IEEE J. Sel. Top. Quantum Electron..

[128] Choi Y.J., Yong S.H., Kim S.J., Hwangbo H., Cho S.M., Pu L.S., Chae H. (2019). Hygroscopic interlayers for multilayer Al_2_O_3_ barrier films. Thin Solid Film..

[129] Jeong E.G., Kwon S., Han J.H., Im H.-G., Bae B.-S., Choi K.C. (2017). A mechanically enhanced hybrid nano-stratified barrier with a defect suppression mechanism for highly reliable flexible OLEDs. Nanoscale.

[130] Yong S.H., Kim S.J., Park J.S., Cho S.M., Ahn H.J., Chae H. (2018). Flexible Carbon-rich Al_2_O_3_ Interlayers for Moisture Barrier Films by a Spatially-Resolved Atomic Layer Deposition Process. J. Korean Phys. Soc..

[131] Park J.S., Yong S.H., Choi Y.J., Chae H. (2018). Residual stress analysis and control of multilayer flexible moisture barrier films with SiN_x_ and Al_2_O_3_ layers. AIP Adv..

[132] Kim H.G., Lee J.G., Kim S.S. (2018). Self-assembled monolayers as a defect sealant of Al_2_O_3_ barrier layers grown by atomic layer deposition. Org. Electron..

[133] Seo S.-W., Jung E., Seo S.J., Chae H., Chung H.K., Cho S.M. (2013). Toward fully flexible multilayer moisture-barriers for organic light-emitting diodes. J. Appl. Phys..

[134] Han Y.C., Jeong E.G., Kim H., Kwon S., Im H.-G., Bae B.-S., Choi K.C. (2016). Reliable thin-film encapsulation of flexible OLEDs and enhancing their bending characteristics through mechanical analysis. RSC Adv..

[135] Wang Z., Chen Z., Wang J., Shangguan L., Fan S., Duan Y. (2023). Realization of an autonomously controllable process for atomic layer deposition and its encapsulation application in flexible organic light-emitting diodes. Org. Express.

[136] Wang Z., Wang J., Li Z., Chen Z., Shangguan L., Fan S., Duan Y. (2023). Crosslinking and densification by plasma-enhanced molecular layer deposition for hermetic seal of flexible perovskite solar cells. Nano Energy.

[137] Chen Z., Wang J., Lin J., Shen Y., Wang M., Duan Y. (2023). Optimizing the gradient stress sandwich structure thin-film encapsulation for super flexible organic light-emitting devices. Appl. Phys. Lett..

[138] Buchwalder S., Bourgeois F., Leon J.J., Hogg A., Burger J. (2023). Parylene-AlO_x_ Stacks for Improved 3D Encapsulation Solutions. Coatings.

[139] Jeong S.Y., Shim H.R., Na Y., Kang K.S., Jeon Y., Choi S., Jeong E.G., Park Y.C., Cho H.E., Lee J.W. (2012). Foldable and washable textile-based OLEDs with a multi-functional near-room-temperature encapsulation layer for smart e-textiles. NPJ Flex. Electron..

